# Phytoplankton ecology in the early years of a boreal oil sands end pit lake

**DOI:** 10.1186/s40793-023-00544-3

**Published:** 2024-01-12

**Authors:** Chantel C. Furgason, Angela V. Smirnova, Joel B. Dacks, Peter F. Dunfield

**Affiliations:** 1 Department of Biological Sciences, University of Calgary, 2500 University Dr. NW, Calgary, AB Canada; 2 Division of Infectious Diseases, Department of Medicine and Department of Biological Sciences, University of Alberta, 116 St. and 85 Ave., Edmonton, AB Canada

**Keywords:** Phytoplankton, Oil sands, Oilsands, Fine fluid tailings, End pit lake, Mine reclamation, Community analysis

## Abstract

**Background:**

Base Mine Lake (BML) is the first full-scale end pit lake for the oil sands mining industry in Canada. BML sequesters oil sands tailings under a freshwater cap and is intended to develop into a functional ecosystem that can be integrated into the local watershed. The first stage of successful reclamation requires the development of a phytoplankton community supporting a typical boreal lake food web. To assess the diversity and dynamics of the phytoplankton community in BML at this reclamation stage and to set a baseline for future monitoring, we examined the phytoplankton community in BML from 2016 through 2021 using molecular methods (targeting the 23S, 18S, and 16S rRNA genes) and microscopic methods. Nearby water bodies were used as controls for a freshwater environment and an active tailings pond.

**Results:**

The phytoplankton community was made up of diverse bacteria and eukaryotes typical of a boreal lake. Microscopy and molecular data both identified a phytoplankton community comparable at the phylum level to that of natural boreal lakes, dominated by *Chlorophyta*, *Cryptophyta*, and *Cyanophyta*, with some *Bacillariophyta*, *Ochrophyta*, and *Euglenophyta*. Although many of the same genera were prominent in both BML and the control freshwater reservoir, there were differences at the species or ASV level. Total diversity in BML was also consistently lower than the control freshwater site, but consistently higher than the control tailings pond. The phytoplankton community composition in BML changed over the 5-year study period. Some taxa present in 2016–2019 (e.g., *Choricystis*) were no longer detected in 2021, while some dinophytes and haptophytes became detectable in small quantities starting in 2019–2021. Different quantification methods (qPCR analysis of 23S rRNA genes, and microscopic estimates of populations and total biomass) did not show a consistent directional trend in total phytoplankton over the 5-year study, nor was there any consistent increase in phytoplankton species diversity. The 5-year period was likely an insufficient time frame for detecting community trends, as phytoplankton communities are highly variable at the genus and species level.

**Conclusions:**

BML supports a phytoplankton community composition somewhat unique from control sites (active tailings and freshwater lake) and is still changing over time. However, the most abundant genera are typical of natural boreal lakes and have the potential to support a complex aquatic food web, with many of its identified major phytoplankton constituents known to be primary producers in boreal lake environments.

**Supplementary Information:**

The online version contains supplementary material available at 10.1186/s40793-023-00544-3.

## Introduction

The Athabasca oil sands in northern Alberta, Canada, is the third largest oil reserve worldwide. In 2018, about 2.8 million barrels of oil were produced per day [1]. Oil extraction from oil sands ore produces oil sands process-affected water (OSPW) and fine fluid tailings (FFT). These waste products contain solids (sand, silt, and clay), caustic salts, residual bitumen, and solvents [2]. The waste is stored in tailings ponds so solids can settle, and the water can be re-used in extraction. Recycling OSPW conserves water but concentrates salts, heavy metals, and acid-extractable organics [3], which are mostly naphthenic acids (NAs). NA concentrations in tailings ponds range from 40 to 120 mg L^−1^, but toxicity can occur in fish at concentrations as low as 2.5 mg L^−1^ [2]. Over 55 years of mining activity, the Alberta oil sands industry amassed over 1.3 trillion L of fluid tailings as of 2020 [4], and continues to produce about 1 billion L of fresh tailings per day [5]. There currently exists no approved large-scale remediation technology for tailings, although projects are underway to test techniques such as tailings filtration, centrifugation, and oxidation [2, 3, 6].

Mined landscapes in Canada are required by law to be reclaimed to an equivalent land capacity to a natural landscape [7]. End pit lakes present a low-cost, long-term land reclamation and remediation strategy applicable on a large scale [3, 8]. An oil sands end pit lake should sequester FFT in a decommissioned open mine pit with an overlying water cap comprised of freshwater and OSPW [3]. Dilution of OSPW via freshwater inputs, biodegradation and tailings consolidation should improve water quality over time [6, 8], eventually allowing integration with surrounding watersheds [8]. End pit lakes are anticipated to become a regular feature of the Athabasca oil sands region, with 23 of these lakes in the planning phase [8]. However, it remains unclear whether end pit lakes will be a suitable method of tailings treatment, with major concerns including contaminant remediation, impacts on wildlife, and their safety for public use, including by Indigenous communities [6, 8]. End pit lakes for metal and coal mines are well-studied, as they have been used for over 100 years and are common around the world [9], with major challenges including acidification and metal/metalloid release [10]. In contrast, research on oil sands end pit lakes is more exploratory and mostly limited to Alberta. The major challenges are very different, and include slow sedimentation, high salinity, and hydrocarbon contamination [6, 8, 11]. Base Mine Lake (BML) is the first and currently the only full-scale demonstration oil sands end pit lake in Northern Alberta [7]. BML is located in a boreal plains ecozone [8] and exhibits a dimictic pattern typical of natural boreal lakes, with mixing occurring in the spring and fall and thermal stratification in the summer and winter [7]. Surface water turbidity increases slightly during turnover as suspended solids are mixed in the water column. The intended land use goal for BML is for it to provide habitat for typical lake plants, macroinvertebrates, and small-bodied fish, but not future public use [7]. The development of BML’s aquatic community will first require the establishment of a phytoplankton community to serve as a food source for higher trophic levels [8].

Phytoplankton are a polyphyletic group of phototrophic cyanobacteria and unicellular eukarya (microalgae) that serve as a primary energy source of aquatic food webs, help drive biogeochemical cycling through carbon, nitrogen, and phosphorous fixation, and contribute dissolved oxygen and organic matter to the ecosystem [12]. The phytoplankton base of boreal lake food webs is typically comprised of low-quality nutrition sources such as cyanobacteria and chlorophytes along with higher quality sources such as diatoms, cryptophytes, dinophytes, and chrysophytes [13, 14]. High quality phytoplankton generally contain more polyunsaturated fatty acids, which higher trophic levels such as ciliates [15, 16], zooplankton, [13] and fish [14] depend on. Cyanobacteria, chlorophytes, and chrysophytes typically increase in boreal lakes during summer and decline in autumn, during which time cryptophytes and diatoms increase [13, 14]. Seasonal or eutrophic algal blooms can increase biological oxygen demand as the produced organic matter decays and consumes O_2_ [12]. Cyanobacterial blooms can be particularly problematic for lake reclamation due to O_2_ depletion during biomass degradation, cyanotoxin production, and the overabundance of poorly-digestible organic matter from some filamentous taxa [8, 17]. This may be a concern for EPLs such as BML because cyanobacterial blooms are common in Alberta boreal lakes [17].

Phytoplankton are impacted by parameters including nutrient availability (predominantly nitrogen and phosphorus), thermal stratification, zooplankton grazing, interspecific competition, and grazing/parasitism by fungi, protozoans, and bacteria [12]. In oil sands tailings environments, phytoplankton may also be inhibited by organic and inorganic contaminants, high salinity, and turbidity [8]. For instance, phytoplankton can show sensitivity to NA concentrations > 6 mg L^−1^ [18, 19]. Despite this, phytoplankton communities can establish in NA-contaminated systems under high nutrient conditions and sufficient light, with primary production comparable to that of natural systems but reduced species diversity [8, 19]. Microcosm studies have suggested that phytoplankton communities similar to non-contaminated sites can establish in systems containing oil sands tailings with a water cap [19]. Numerous phytoplankton taxa are tolerant to high NA concentrations (> 30 mg L^−1^), including some in the *Chlorophyta*, *Euglenophyta*, and *Synechococcaceae* [20]. Prior to water capping, BML was a tailings pond known as West-in Pit and had very low eukaryotic diversity, perhaps due to high toxicity (from NAs, heavy metals, and high salinity), high turbidity, and low O_2_ content [21]. The eukaryote community was fungi-dominated, with only very few *Euglenophyta*, *Chrysophyceae*, and *Chlorophyta* phytoplankton based on 18S rRNA gene analysis [21]. In 2015, three years after water capping, 18S rRNA gene sequencing analysis revealed that only 6% of the reads belonged to exclusively phototrophic phyla, while 27% belonged to phyla containing both heterotrophs and phototrophs [22].

The phytoplankton community in BML will be crucial to its development. We used high-throughput PCR amplicon sequencing of the 23S, 18S, and 16S rRNA genes to investigate the phytoplankton community in BML over 5 years from 2016 to 2021. We hypothesized that BML’s phytoplankton community would initially resemble those in active tailings ponds but become more akin to a freshwater system over time as water quality improves in the water cap. We also quantified algae based on quantitative PCR of the 23S rRNA gene, phytoplankton cell counts, biomass, and chlorophyll *a* content. We predicted that the abundance of phytoplankton in BML would increase over time due to improvements in water quality and clarity.

## Materials and methods

### Site descriptions and sampling

BML (57.0109°N, 111.6219°W) is 8 km^2^ in area, located about 45 km north of Fort McMurray, Alberta, Canada (Additional file 1: Fig. S1). Prior studies offer comprehensive descriptions of BML [7, 23]. The site was first commissioned in 1978 as a mining pit called West-In Pit (WIP), then converted to a tailings pond in 1994. In 2012, FFT were added to a depth of 45–50 m and capped with 5 m of fresh water and OSPW. The capped system was renamed Base Mine Lake (BML). BML is isolated from the local watershed and the water level is maintained at 308.7 m above sea level via pump-in of freshwater from Beaver Creek Reservoir (BCR) or pump-out to the nearby extraction plant. BML receives inflow from rain, snow, and runoff, but pump-in from BCR is the major input source [7], contributing ~ 4–9% of water cap volume from 2016 to 2019, with no inflow in 2021 (see Additional file 1: Table S1) [7]. As of 2021, the water cap depth increased to ~ 10–13 m due to FFT settling and dewatering with a water cap volume of ~ 71 Mm^3^. BCR was used as a freshwater control site in our study: it does not contain any tailings or OSPW but is immediately adjacent to BML and contains some of the same freshwater.

BML is dimictic, with ice-off in April to early May, spring turnover from May to mid-June, summer stratification mid-June to early September, fall turnover in September, and ice-on in mid-November. Turbidity and total suspended solids are highest during turnovers in the spring and fall and lowest in the summer, with water temperatures ranging from 0 to 24 °C depending on season and depth. From 2012 to 2016, water turbidity (50–350 Nephelometric Turbidity Units; NTU) was about ten times higher than turbidity in local freshwater bodies due to clay suspension from FFT. In an effort to sequester the clays, the chemical coagulant aluminum potassium sulfate (alum) was added to BML from September to October of 2016. Turbidity immediately decreased and has remained lower than pre-2016 values in BML every year since (ranging from 4 to 28 NTU in 2021) [7]. Surface water quality in BML has also improved gradually over time. As of 2016, dilution with freshwater has reduced metal ion concentrations in BML to within water quality guidelines, although salinity is still 10 times higher than Athabasca River water [24]. Petroleum-associated compounds including total phenolics, F2 hydrocarbons, and NAs remain elevated compared to natural sources, with average NA concentrations ranging from 27 to 30 mg L^−1^ in 2021 [7], compared to < 1 mg L^−1^ in the Athabasca River [2].

The neighboring water bodies Beaver Creek Reservoir (BCR) and Mildred Lake Settling Basin (MLSB) served as controls for an artificial freshwater ecosystem and an active tailings pond, respectively. BCR, located immediately south of BML (Additional file 1: Fig. S1), is 2.2 km^2^ in size with a maximum depth of ~ 10 m and a mean depth of 2.2 m. Unlike BML, only weak thermal stratification is observed in BCR in the summer and turbidity is lower, ranging from 1 to 19 NTU. Phytoplankton, invertebrates, and fish are abundant in the reservoir. See Additional file 2: Table S1 for comparison of the physicochemical parameters in BML and BCR over years. MLSB is an active tailings pond ~ 10 km^2^ in size located just north of BML. It is the oldest and largest tailings pond in the region and is characterized by an active methane cycle [3].

BML samples were taken at 1–4 week intervals during the ice-off period (May–October) and via ice coring in the winter (February–March) from three fixed sampling platforms (Additional file 1: Fig. S1) as described previously [23]. The three sampling platforms were treated as replicates. BCR and MLSB samples were taken less frequently, at about 1-month intervals. BCR samples were taken from three different shoreline sampling sites and MLSB samples were taken from a single shoreline sampling site. All samples were taken from the surface (0.3–0.6 m) using Van Dorn samplers. Samples were shipped in polypropylene bottles on ice to the University of Calgary (c. 2 d shipping time) then stored at 5–8 °C upon delivery, typically for 1–2 d until processing. The hold time before sample processing likely influenced microbial communities to some extent. However, studies have shown that keeping samples on ice prior to DNA extraction is among the most effective controls for maintaining microbial community integrity [25], and that samples kept on ice for 3 days were similar to day 0 samples in terms of their microbial community composition [26]. A major wildfire delayed sampling in 2016 until the end of June. Sampling was also interrupted in 2020 due to a pandemic shutdown.

### Molecular community analyses via gene amplicon sequencing

Water samples (500 mL) were processed via centrifugation with DNA extracted from the pelleted material as described previously [23]. Phytoplankton were identified using PCR amplicon sequencing with primers targeting the 23S (V5), 18S (V4), and 16S (V3-4) rRNA genes (Additional file 2: Table S2). The 23S rRNA priming sequences are present only in plastids and cyanobacteria [27], those for the 18S rRNA gene are universal to eukaryotes, including eukaryotic phytoplankton [28], and those for the 16S rRNA gene are universal to bacteria, including cyanobacteria and chloroplasts [29]. Each primer had Illumina adaptors attached to the 5’ end (Forward 5’-TCG TCG GCA GCG TCA GAT GTG TAT AAG AGA CAG-3’; Reverse 5’-GTC TCG TGG GCT CGG AGA TGT GTA TAA GAG ACA G-3’). PCR amplification conditions for each primer set are given in Additional file 2: Table S3. Amplicon libraries were prepared using MiSeq Reagent Kit v3 with 600 cycles (Illumina part number MS-102–3003) as described in [23]. Each MiSeq lane had ~ 400 pooled amplicons. Feature tables from various lanes were merged for final analyses. Additional file 2: Table S4 lists sample metadata (i.e., source, year, season).

Sequencing data were analyzed with Quantitative Insights Into Microbial Ecology 2 (QIIME2) version 2021.4 [30]. Cutadapt software was used to trim primers and Illumina adaptor sequences from all fastq files [31]. The software package DADA2 was used to denoise, pair reads, and remove chimerae [32], and a quality score was assessed for 10 random samples per run to adjust denoising parameters. Taxonomy was assigned to each Amplicon Sequence Variant (ASV) using the feature-classifier plugin [33] with a naïve Bayes classifier approach. Taxonomy for the 23S rRNA gene was assigned using the MicroGreen database (μgreen-db) [34], and taxonomy for the 16S and 18S rRNA genes were assigned through the SILVA 138 database [35]. As described in Additional file 2: Tables S5-S6, the 16S and 18S rRNA gene datasets were filtered to remove non-phytoplankton sequences, and all three datasets were filtered to remove taxa unassigned at the phylum level. Additional file 2: Table S5 describes how reads were truncated for each primer set and taxonomy filtering parameters. Additional file 2: Table S6 indicates the number of features and reads for each filtration step and rRNA gene dataset. The identities of the major ASVs belonging to key phytoplankton genera were verified using the online SILVA Alignment, Classification, and Tree (ACT) service (https://www.arb-silva.de/aligner/) [36] and NCBI BLAST (https://blast.ncbi.nlm.nih.gov/) [37] (Additional file 2: Tables S7). This resulted in some manual taxonomic reassignments (See Additional file 1: Note 1 and Additional file 2: Tables S8–S10).

### 23S-rRNA gene based quantitative PCR

Quantitative PCR (qPCR) of the 23S-rRNA gene was performed as described in [27] to quantify phototrophs. qPCR was done in reactions containing 1 µL of sample gDNA, 1 µL of each forward/reverse primer (1.25 µM each), 5 µL of SYBR Green ssoAdvanced PCR Mix (Qiagen, Venlo, Netherlands), and DNAse-free water up to 10 µL (Qiagen, Venlo, Netherlands) on a Rotor-Gene 6000 thermocycler (QIAGEN, Venlo, Netherlands). Based on a search of the MicroGreen 23S rRNA gene database, the 23S rRNA gene primers used were determined as universal to phytoplankton, matching 1942 of 2326 total database sequences [34]. Standards were constructed from a *Cryptomonas* 23S rRNA gene sequence, which was PCR-amplified as described above from BML water samples and then cloned into a PJET 3.0 plasmid (ThermoFisher, Waltham, MA, USA) based on the CloneJET PCR Cloning Kit protocol (Thermo Scientific). The plasmids were PCR-amplified using the 23S rRNA gene primers, then amplicons were quantified with a Qubit HS kit (Invitrogen, Carlsbad, CA, USA) and serially diluted over 7 orders of magnitude. The detection limit was ~ 100 gene copies mL^−1^ and median amplification efficiency was 89%. Examination of qPCR melt curves suggested primer specificity.

### Microscopy analyses

Samples for cell counts (cells L^−1^), biomass (mg m^−3^), and chlorophyll *a* (µg L^−1^) were collected using Van Dorn units. Samples were taken from the euphotic zone (0.6–0.8 m) or immediately under the ice. BML samples were taken from three platforms, but BCR samples were only taken from one of the three sampling sites. Three pseudo-replicates of two combined grabs were taken from each platform for a total of 500 mL per sample. Phytoplankton samples were preserved with approximately 15 drops of Lugol’s solution and then sent to the EcoAnalysts laboratory (Moscow, ID, USA) for taxonomic identification and enumeration. A 5–25 mL aliquot was extracted from each sample and placed into an Utermöhl counting chamber. The transect method was used to enumerate phytoplankton identified to the lowest practical taxonomic level (LPL), with at least 300 units counted per sample. Units were counted as single cells, filaments, or colonies depending on the distribution of phytoplankton. The biovolume (μm^3^) of each phytoplankton LPL was estimated from mean dimensions measured at 630 × magnification and related to geometric shapes [38]. Biovolume measurements were calculated once for each taxon that represented < 5% relative abundance in the sample and 10 times for each that represented > 5%. For taxa with great discontinuities or variations in size, at least 20 biovolume measurements were calculated per sample. Mean biovolumes were calculated for each taxon based on the quantity of individuals within each colonial taxon. Mean cell biovolume (μm^3^) was converted to biomass for all individual phytoplankton taxa assuming a specific gravity of 1 (i.e., 1 µm^3^ = 1 µg). Average biovolumes and (ranges) in μm^3^ cell^−1^ were as follows: *Euglenophyta* 12,810 (77 to 257,066), *Cryptophyta* 12,398 (19 to 661,200), *Bacillariophyta* 2323 (57 to 226,195), *Chlorophyta* 1882 (71 to 117,718), *Chrysophyceae* 500 (18 to 3534), and *Cyanobacteria* 270 (18 to 4110). Each taxon’s total sample biomass (wet weight) was calculated using the equation: Total Biomass (µg L^−1^) = Average Biomass (µg cell^−1^) x Total Abundance (cells L^−1^).

Chlorophyll *a* concentrations (µg cm^−2^) were measured at the University of Alberta’s Biogeochemical Analytical Service Laboratory (BASL) in Edmonton, Alberta using fluorometric analysis [39]. Detection limits for cell count, biomass, and chlorophyll *a* measures were, respectively, 20 cells L^−1^, 0.001 mg m^−3^, and 0.50 µg L^−1^.

### Community analyses

#### Molecular data

Heatmaps were constructed in the RStudio software using the packages *gplots* and *vegan* and the heatmap.2 function with the Bray–Curtis clustering algorithm and average linkage hierarchical clustering [40]. Alpha-diversity statistics were calculated at the ASV level in R for Chao1, observed genera, and the Shannon index using the package *otuSummary*. The package *SRS* (Scaled with Ranked Subsampling) was used to normalize samples at the ASV level [41].

#### Molecular and microscopy data

Nonmetric multidimensional scaling (NMDS) ordinations were performed in R with the package *vegan* [40] and taxa tables were normalized using *SRS* [41]. The Bray–Curtis index was used for the dissimilarity measure with 10,000 iterations. Analysis of Similarities (ANOSIM) was performed in R to test whether variation in community composition within sites was greater than across-site variation [42]. Significant associations between ASVs and site variables were tested using Indicator Species Analysis (ISA) in R with the package *indicspecies*, which detects whether certain taxa drive differences in community composition across sites. Optimal indicator species are defined as occurring exclusively with high frequency within a given site. Indicator ASVs were computed (p < 0.05, 10,000 permutations) using the functions multipatt and IndVal.g, which accounts for unbalanced across-group sizes [43]. Species indicator P values were adjusted for multiple testing with the Benjamini–Hochberg method (p-value < 0.01 unless indicated otherwise) [44].

Palmer’s algal genus pollution index [45] uses phytoplankton as bioindicators of organic pollution, and is used to evaluate the water quality of freshwater environments (e.g., [46]). This index was developed for microscopic count data, but we combined both molecular and microscopic data for more comprehensive taxonomic coverage. We calculated this index by scoring phytoplankton genera with average relative abundance of ≥ 0.50% and occurring in at least 3 samples for that given year and source for at least one dataset (calculations given in Additional file 2: Table S11). This index was used to gauge ecological status in BML over time and to compare BML and BCR based on genera known to be bioindicators of organic pollution, with the caveat that it has limited utility and provides only a rough estimate for comparing water quality in BML over time and between BML and BCR.

## Results

### Phytoplankton community composition at the phylum level

**Fig. 1:**
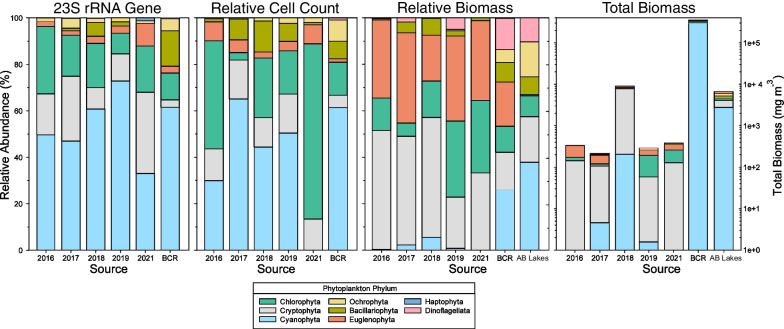
Relative abundances (%) of the major phytoplankton phyla in surface waters of BML (0.3–0.6 m for 23S rRNA gene data and 0.6–0.8 m for cell count and biomass data) for each sampling year, shown in comparison to the freshwater reservoir BCR (averaged for all years) and to relative biomass estimated in Alberta boreal headwater lakes [47]. Total biomass (mg m^−3^) is also given as a logarithmic scale. For direct comparison of the different sites, only samples from July through September were included

Community compositions based on 23S rRNA gene amplicon sequencing, microscopic cell counts, and biomass for the years 2016–2021 during the months of July to September are presented in Fig. 1, and compared with the average phytoplankton biomass community composition of Alberta boreal headwater lakes reported in [47]. Compositions are similar across the three measurements despite some obvious biases. Firstly, cell count and biomass methods only identified phytoplankton that are morphologically distinguishable and therefore did not identify certain coccoidal picophytoplankton identified in sequencing such as the chlorophyte *Choricystis* or the cyanobacterium *Synechococcus* (see Additional file 1: Note 2 for list of phytoplankton counted in microscopy)*.* This may partially explain the generally higher counts of cyanobacteria based on molecular analyses. The small cyanobacteria were less important in biomass measurements than in cell or gene counts, while euglenophytes and cryptophytes were more important due to their large cell sizes. Overall, all phytoplankton phyla except for *Haptophyta* occurred in similar proportions between 23S rRNA gene and cell count data, whereas biomass was more discrepant.

Based on the 23S rRNA gene amplicon and cell count analyses, the phyla with the greatest relative abundances in BML were *Cyanobacteria* (0–73%), *Chlorophyta* (3.3–75%), and *Cryptophyta* (9.2–35%), with lower proportions of *Euglenophyta* (1.9–9.7%), *Bacillariophyta* (0.10–13.3%), and *Ochrophyta* (0–4.3%) (Fig. 1). The BCR community was also predominantly *Cyanobacteria* (61%) and *Chlorophyta* (14–15%) but had greater proportions of *Bacillariophyta* (7.5–15%) and less *Cryptophyta* (3.2–5.2%) than BML. Compared to natural Alberta boreal lakes, BML’s biomass estimates were similar for *Cyanophyta*, *Cryptophyta*, *Bacillariophyta*, and *Chlorophyta* (Fig. 1). *Euglenophyta* had much lower relative abundance in boreal lakes (< 0.5%) compared to BML, while *Ochrophyta* and *Dinoflagellata* were higher in boreal lakes (15% and 11%, respectively). Haptophytes, which were not reported by the boreal lake study [47], occurred in small amounts in BCR for all years (0.13%), and appeared in BML beginning in 2017 (0.004–1.3%). Median and mean values for phyla for all samples and years are presented in Additional file 2: Table S12 and phyla compositions for each season are presented in Additional file 1: Fig. S2.

### Genus-level diversity

**Fig. 2:**
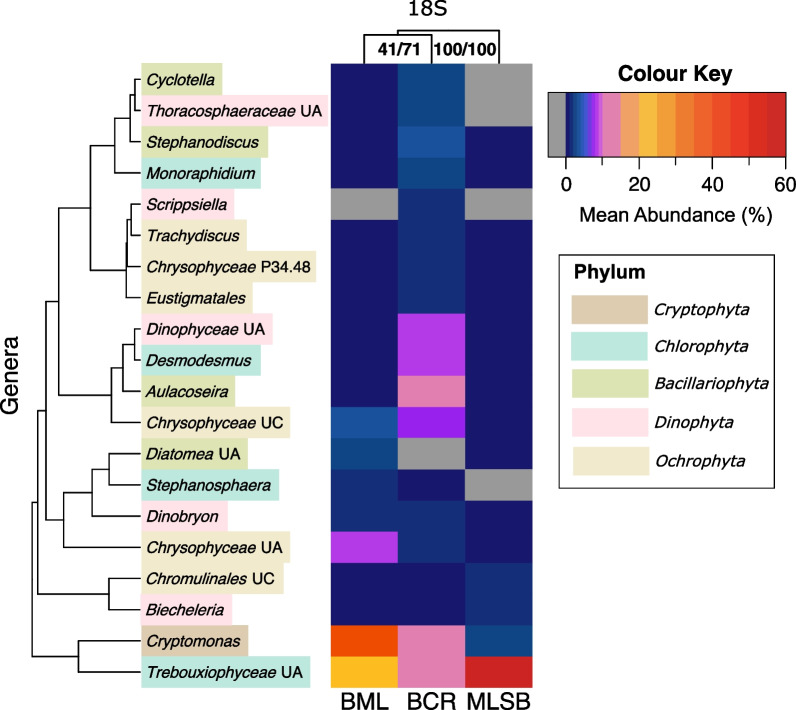
Heatmap with double hierarchical clustering analysis, depicting relative abundances of genera that comprise ≥ 1% of the total detected in the 18S rRNA gene sequencing analysis for shared sample dates between BML, BCR, and MLSB, normalized to 1000 counts using the R package *SRS*. Genera are colour-coded by phylum and clustered based on similarities across sites. Hierarchal cluster analyses of sites shown at the top of the heatmap include approximately unbiased alpha levels (AU) (p-values computed by multiscale bootstrap resampling) and bootstrap probability for 1000 resamplings (BP) of each node (AU/BP). AU values > 95 indicate significant cluster nodes. BML and BCR consistently cluster apart from MLSB

Summarizing the rRNA gene sequencing data, four highly abundant phytoplankton genera (≥ 5% average relative abundance in at least 2 rRNA gene sequencing datasets) were identified in BML: *Synechococcus* (*Cyanobacteria*), *Choricystis* (*Chlorophyta*), *Cryptomonas* (*Cryptophyta*), and *Euglena* (*Euglenophyta*) (Additional file 1: Figs. S3-4). Additionally, *Prochlorococcus* (*Prochlorophyta*; grouped with *Cyanobacteria*) and *Planktothrix* (*Cyanobacteria*) had high relative abundance in BML based on the 23S rRNA gene sequencing. Genera that had high relative abundance in the natural control site BCR included *Planktothrix*, *Synechococcus*, *Aulacoseira* (*Bacillariophyta*), *Cryptomonas*, and *Desmodesmus* (*Chlorophyta*), while those for the tailings pond control MLSB were *Prochlorothrix* (*Cyanobacteria*), *Prochlorococcus*, *Chlorella* (*Chlorophyta*), and *Nannochloropsis* (*Ochrophyta*). A number of genera with lower relative abundance were also detected in each site. Based on the 18S rRNA gene sequencing analysis (which contained the most shared sample dates between sites), the overall community detected in BML was more similar to the freshwater reservoir BCR than the tailings pond MLSB (Fig. 2). For further details of the genera detected in each site, see Additional file 2: Table S13.

**Fig. 3:**
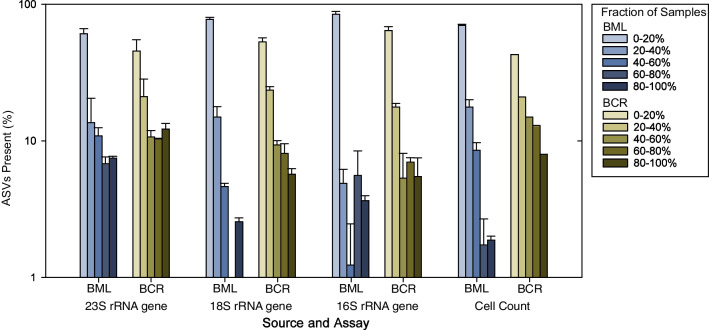
Taxa occurring in different proportions of surface water samples taken on shared sample dates between BML and BCR for gene sequencing data (0.3–0.6 m depth) and cell count data (0.6–0.8 m depth) over all years. Taxa for the gene sequencing data were ASVs, while those for the cell counts were species. For each set of bars, the lighter shades indicate ephemeral taxa (present in few samples), while the darker shades indicate increasingly more persistent taxa (present in most samples). BML showed more ephemeral and fewer persistent taxa than BCR. Data were normalized using scaling with ranked subsampling (SRS) [41] to 4000, 1000, 100, and 10,000 for the 23S, 18S, and 16S rRNA gene datasets and cell count data, respectively. Each bar represents the mean of three replicates ± 1 SEM. No replicates were available for BCR cell count data

Based on relative abundance data for the molecular analyses, all of the four most abundant genera in BML were persistent, occurring in ≥ 75% of all samples analysed (Additional file 2: Table S14A) [48]. Genera persistent in all three sources based on at least one gene sequencing analysis included *Chlorella*, *Choricystis*, *Prochlorococcus*, *Prochlorothrix*, *Synechococcus*, *Euglena*, and *Nannochloropsis*, while *Cryptomonas* and *Chlorella* were persistent in both BML and BCR. BCR contained a greater percentage of persistent taxa for each analysis compared to BML (Fig. 3, Additional file 2: Table S14B), i.e., many taxa in BML were more transitory.

### Site comparisons

**Fig. 4:**
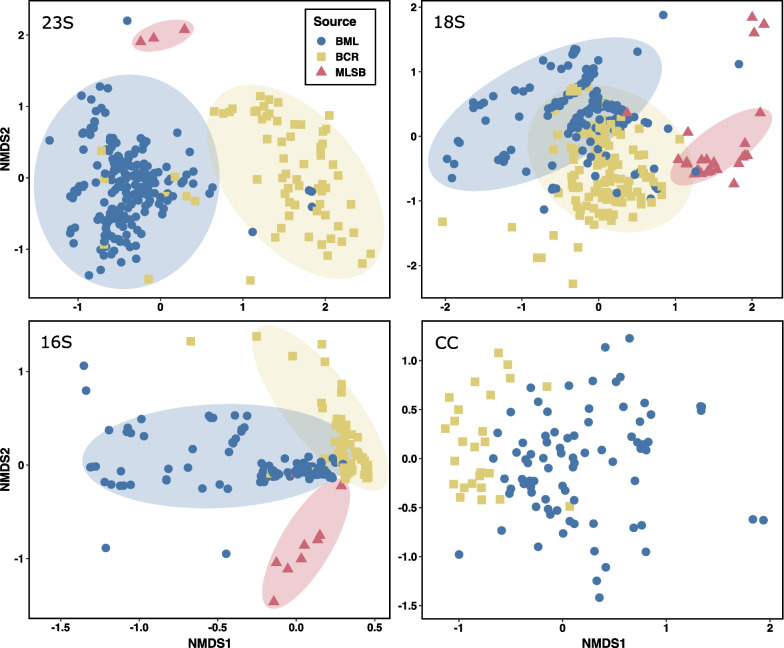
Non-metric multi-dimensional scaling ordination (NMDS) plots in BML, BCR, and MLSB surface waters based on Bray–Curtis dissimilarities of phytoplankton communities. Data were normalized using scaling with ranked subsampling (SRS) [41] to 4000, 1000, 100, and 10,000 for the 23S, 18S, and 16S rRNA gene and cell count datasets, respectively. Taxa were classified at the ASV-level for molecular data and at the species-level for cell count data. k = 2 axes for all plots. Stress scores were 0.198, 0.207, 0.142, and 0.239 for the 23S, 18S, and 16S rRNA gene and cell count datasets, respectively. Clusters with ANOSIM support are indicated by coloured circles; all samples were included in ANOSIM analysis, but circles were hand-drawn to emphasize clustering (See Additional file 2: Table S15). Winter samples were not included in this analysis as there was no winter sampling for BCR

Community composition had greater variation between sources than within sources based on ANOSIM (Additional file 2: Table S15), and distinct clustering by source was evident in NMDS plots using each of the ASV-level molecular data and the cell counts (Fig. 4). Therefore, BML’s phytoplankton community composition was usually distinct from both control sites over the entire study.

Indicator Species Analysis (ISA) was performed on normalized datasets for molecular and cell count data (Additional file 2: Table S16) to identify genera and ASVs significantly indicative of a given source over the entire 5 years (stat > 0.70, *p* < 0.01). In order to normalize comparisons, this analysis used sample dates where all three sites were sampled and each sample was normalized to an equal number of sequence reads (4000, 1000, and 100 for the 23S, 18S, and 16S rRNA genes, respectively). In comparing BML to BCR using matched sample dates, the only genera indicative of BML were *Oocystis* (*Chlorophyta*) and *Cryptomonas* (*Cryptophyta*), based on cell count data and the 16S rRNA gene data, respectively. In contrast, *Dolichospermum* (*Cyanobacteria*; formerly known as *Anabaena*), *Planktothrix* (*Cyanobacteria*), *Aulacoseira* (*Bacillariophyta*), *Desmodesmus* (*Chlorophyta*), *Monoraphidium* (*Chlorophyta*), and *Trachydiscus* (*Ochrophyta*) were indicative of BCR based on multiple analyses. *Cryptomonas* was indicative of both BML and BCR based on the 18S rRNA gene. Indicator genera for MLSB were *Chlorella* (*Chlorophyta*) and *Tetradesmus* (*Chlorophyta*). See Fig. 2 and Additional file 1: Fig. S4 for a qualitative representation of these indicator genera.

Samples were also analyzed at the ASV level to determine whether different strains of the same genus or species were characteristic of each site (Additional file 2: Tables S17-S20). Although the same genera frequently occurred in 2 or 3 sites, these genera were usually represented by different site-specific ASVs based on ISA (Additional file 2: Table S17). For example, different *Cyanobium*/*Synechococcus* and *Cryptomonas* ASVs were found to be indicative of BML versus BCR. Similarly, different *Prochlorococcus* and *Prochlorothrix* ASVs were found to be indicative of BML versus MLSB.

To illustrate this ASV-site specificity in more detail, we list the top ten most abundant ASVs that were exclusive to BML or BCR in Additional file 2: Table S18. ASVs were defined as exclusive if they were not detected in the other source for all samples considered. The detection limit was 1 read in a total of 4000, 1000, 100, and 10,000 normalized reads for the 23S, 18S, and 16S rRNA genes and cell count data, respectively. All of the genera *Prochlorococcus*, *Synechococcus*, and *Chlorella* had certain ASVs exclusive to either BML or BCR for the 23S rRNA gene. More broadly, the top ten most abundant, not necessarily exclusive, ASVs for each of *Choricystis*/*Picochlorum*, *Cryptomonas*, *Synechococcus*/*Cyanobium*, *Euglena*, *Prochlorococcus*, and *Prochlorothrix* (Additional file 2: Table S19) include some that were shared between BML and BCR, but others that were clearly more dominant in one site. In particular, only one of the top 10 *Prochlorococcus* ASVs was shared between BML and BCR for the 23S rRNA gene sequencing. The most dominant *Cryptomonas* ASV in BML was 8.3 times less abundant in BCR. Four shared, abundant ASVs are plotted over time (in Additional file 1: Fig. S5) to illustrate two different patterns observed. Those in (A-C) appear in both BML and BCR but have higher relative abundances in BML and persist even during periods when they are virtually absent in BCR, such as in 2021, when no inflow from BCR to BML occurred. This suggests that certain strains have established in BML and exhibit their own growth patterns in this site over time, without the need for continued inoculation from BCR. In contrast, Additional file 1: Fig. S5 (D) shows a *Synechococcus* ASV that is abundant in BCR but does not establish in BML, although other strains of the same species do.

In summary, these data suggest that although many of the same genera and ASVs (strains) occur in 2–3 sites, there is a distinct site-specific microdiversity. Even though many more samples (and total sequences) of BML were processed, more ASVs were exclusive to BCR for gene sequencing data, indicating that the highest strain diversity was found in the freshwater site (Additional file 2: Table S20). The conditions in BML select for the growth of particular strains, and the community in BML does not simply reflect the community in BCR that is added via BCR pump-in water.

### Changes over time

BML showed some weak clustering of community compositions by year based on NMDS analyses (Additional file 1: Fig. S6, Additional file 2: Table S15), but there was a lot of overlap across years, particularly for the 23S rRNA gene sequencing. Thus, long-term trends were suggested but were obscured by shorter-term variability when looking at entire communities.

**Fig. 5:**
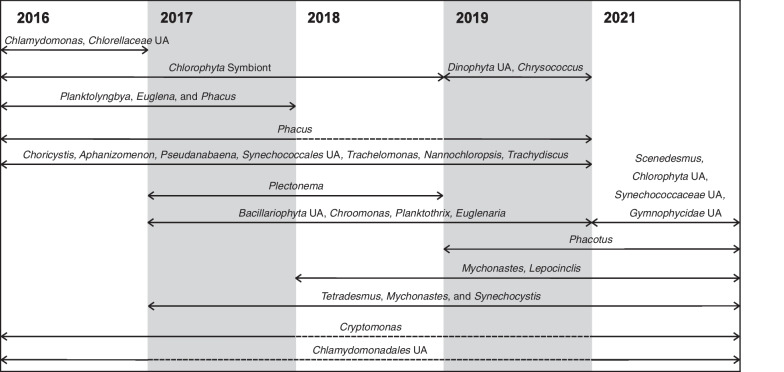
Summary of genus-level Indicator Species Analysis (ISA) results for BML surface waters over 6 years. Gaps are given by dashed lines to show that a genus was not indicative for that year (although most genera were always present at some level). UA stands for unassigned at any taxonomic level below the taxon shown. For details on the ISA statistics, see Additional file 2: Table S21. No sampling was conducted in 2020

Therefore, indicator species analyses (ISA) were performed for BML by year to complement relative abundance data (BCR was not included in this analysis; Fig. 5, Additional file 2: Table S21). The community was dynamic over the 5-year study, with some genera declining and others increasing over 2–3-year periods. For example, there was a general increase in relative abundances of some genera of *Chlorophyta*, *Cyanobacteria*, *Euglenophyta*, *Ochrophyta*, and *Bacillariophyta* (compared to other microbes) from 2016 to 2019, but a slight decline in 2021. Conversely, dinophytes and haptophytes increased in later years (Fig. 5). A major change was the decline in *Choricystis* abundance in 2021, although the overall *Chlorophyta* fraction of the community did not decrease (Additional file 1: Fig. S7). ISA results for the 23S and 18S rRNA genes also showed *Choricystis* was an indicator for the BML community for 2016–2019, while *Mychonastes* was indicative of 2018–2021 (Additional file 2: Table S21). The 23S and 18S rRNA gene datasets each contained a single unassigned *Chlorophyta* ASV with a very high number of reads in BML for 2021 and low reads for previous years. Detailed analyses indicated that this represented an uncultured *Oocystis* (see Additional file 1: Note 3 for more detail).

### Seasonality

**Fig. 6:**
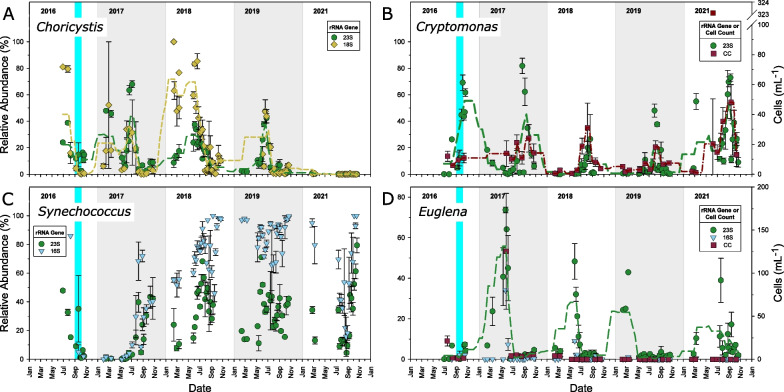
Relative abundances of the four major algal genera in BML surface waters: **A** *Choricystis*, **B** *Cryptomonas*, **C** *Synechococcus*, and **D** *Euglena*, based on 23S, 16S and/or 18S rRNA gene amplicon sequencing. Non-cyanobacteria were filtered from the 16S rRNA gene dataset and known non-photosynthetic eukaryotes were filtered from the 18S rRNA gene dataset. Also included for B) and D) are microscopic cell count data. Note that the different analyses are not fully comparable due to their respective limitations and biases (see Discussion: Gene Sequencing and Microscopy Complementarity). Data points are means of three platforms ± 1 SEM. Where error bars are not seen they are contained within the symbol. The teal bar indicates alum addition. Dashed lines indicate running averages calculated with smoothing in SigmaPlot, in which a running average is based on 10 samples. In panel B, the single extreme outlier in the microscopic count data was not used in the running average

Sequencing, cell count, and biomass data indicated similar seasonal patterns in overall community composition (Additional file 1: Fig. S2). However, these patterns were best illustrated by molecular analyses, which were the least time-consuming analyses and hence could be applied to more samples over time (Fig. 6). Three of the four most abundant phytoplankton genera, *Cryptomonas*, *Choricystis*, and *Euglena,* showed consistent seasonality over the course of the study, with abundance peaks at similar times each year. *Cryptomonas* generally peaked during August to September, around the time of late summer stratification and fall mixis. *Synechococcus* was more variable, but also usually peaked during the summer or early autumn. *Choricystis* peaked in June or July (the lack of a peak in 2021 could have been due to a lack of sampling) and *Euglena* in March to July. Based on ISA (Additional file 2: Table S22) and time-course data (Additional file 1: Figs. S7-S13), several less abundant genera also exhibited seasonal patterns, summarized in Additional file 2: Table S23.

### Alpha diversity

**Fig. 7:**
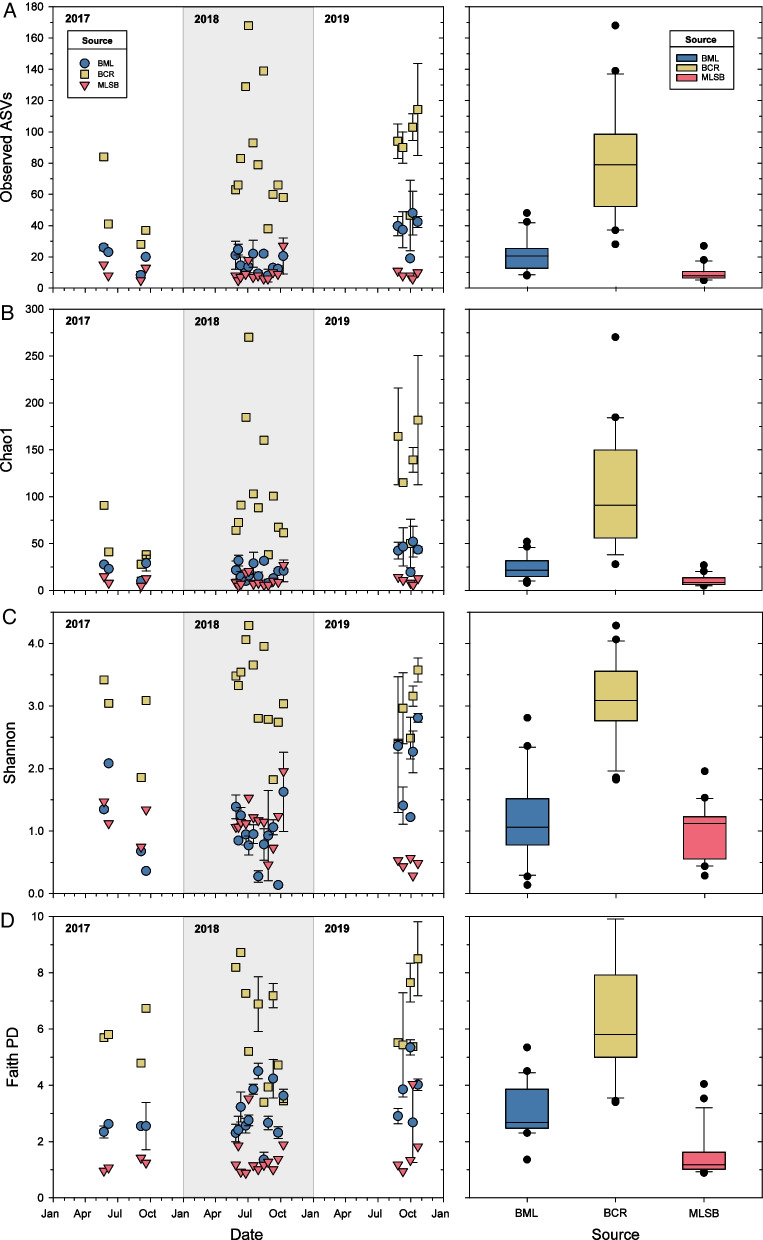
Alpha-diversity indices in BML, BCR, and MLSB surface waters on shared sample dates, based on 18S rRNA gene sequencing at the ASV level, normalized to 1000 counts and filtered to include only phytoplankton. The 18S rRNA gene was chosen because it had the most shared sample points. Data points in the left panels are means of three samples ± 1 SEM, except for MLSB samples, which were unreplicated. Stem-and-leaf plots (right panels) for each index indicate means, 95% confidence intervals, and ranges averaged by site over all sampling dates; points indicate outliers. Diversity indices were significantly different across sites in all cases except in BML versus MLSB for the Shannon diversity index

Using only shared sample dates (i.e., times when all three sites were sampled), α-diversity in BML was intermediate between BCR and MLSB based on the 18S rRNA gene sequencing. The ASV data are shown in Fig. 7, genus level data are shown in Additional file 1: Fig. S14. BML was significantly less diverse than BCR for all indices based on a MANOVA with date and site as factors (Tukey’s HSD, *p*-value < < 0.01; Additional file 2: Table S24), and significantly more diverse than MLSB for all indices except Shannon (Tukey’s HSD, *p*-value < 0.01; Additional file 2: Table S24). Hence, several measures of α-diversity in BML were intermediate to the two control sites.

Similarly, in the 23S rRNA gene dataset, BML was significantly less diverse than BCR at the ASV level for observed ASVs and Faith’s index based on two-way MANOVA with date and site as factors (Additional file 1: Fig. S15; Tukey’s HSD, p < 0.01; Additional file 2: Table S24). Only BML and BCR were compared in the 23S rRNA gene dataset analyses because too few MLSB samples were available.

Trends over time were generally neutral for each diversity index and gene sequencing dataset, suggesting that alpha diversity was quite stable in BML over 5 years (Additional file 1: Figs. S15, S16, Additional file 2: Table S25). Slightly negative trends over time in BML were observed for diversity indices in the complete 23S rRNA gene dataset that accounted for both bacteria and eukarya (Additional file 1: Fig. S15; Additional file 2: Table S25). These likely reflected changes in cyanobacteria, as no such trends were evident in eukaryote-only datasets (18S rRNA and 23S rRNA filtered to remove bacteria). The only significantly positive temporal trends in the complete 23S rRNA gene dataset were for the Shannon and Faith indices in BCR, which were small (slopes of + 0.03 and + 0.15 per year, respectively). In the eukaryote-only datasets, the 23S rRNA gene diversity results had no significant upward or downward changes over time, and only slight positive changes were observed in the 18S rRNA gene dataset (Additional file 1: Fig. S16 and Additional file 2: Table S25). Both the weakly negative regression lines in Additional file 1: Fig. S15 and the weakly positive regression lines in Additional file 1: Fig. S16 explained very little of the overall variability (r^2^ = 0.033–0.11), and were likely influenced by a few outliers, making any non-zero trends tentative.

### Palmer’s pollution index

Palmer’s pollution score was calculated for BML for each separate year and for BCR as an average over all years (Table 1). BML’s pollution score ranged from low to high (11–26) over the five years but was in the high range for most years and did not change significantly over time (regression, p-value = 0.92). BCR’s pollution score was in the low to moderate range (11–17) with no significant change over time (regression, p-value = 0.91), and was significantly lower compared to BML’s pollution score (Tukey’s HSD, p-value = 0.038).

**Table 1:** Palmer’s Pollution Index results for BML and BCR surface waters (≤ 0.6 m) by year and overall

Phytoplankton Genus	Index Value	BML						BCR					
		2016	2017	2018	2019	2021	Total	2016	2017	2018	2019	2021	Total
*Cyclotella*	1	–	18S	–	–	–	18S	–	23S, 18S, 16S	23S, 18S, CC	23S, 18S, 16S, CC	23S	23S, 18S, 16S, CC
*Gomphonema*	1	–	NA	–	–	–	–	–	–	–	CC	–	CC
*Melosira*	1	NA	NA	NA	NA	NA	NA	NA	NA	NA	NA	NA	NA
*Navicula*	3	–	CC	CC	CC	–	CC	–	–	–	–	CC	CC
*Nitzschia*	3	–	CC	CC	CC	–	CC	–	18S, CC	–	–	–	18S, CC
*Synedra*	2	–	–	–	–	–	–	–	–	–	CC	–	CC
*Ankistrodemsus*	2	–	–	–	–	–	–	–	–	–	–	NA	–
*Chlamydomonas*	4	23S, CC	23S	–	–	–	23S, CC	CC	–	–	–	–	CC
*Chlorella*	3		23S	23S	23S, 18S	23S	23S, 18S	CC	23S	23S	23S	23S	23S, CC
*Closterium*	1	–	NA	–	–	NA	–	NA	–	–	–	NA	CC
*Micractinium*	1	NA	–	NA	NA	NA	–	–	NA	–	–	NA	–
*Pandorina*	1	NA	NA	CC	CC	CC	CC	NA	NA	NA	–	NA	–
*Scenedesmus*	4	–	18S	CC	18S	18S, CC	18S, CC	CC	CC	CC	18S	–	18S, CC
*Stigeoclonium*	2	NA	NA	NA	NA	NA	NA	NA	NA	NA	NA	NA	NA
*Anacystis*	1	NA	NA	NA	NA	NA	NA	NA	NA	NA	NA	NA	NA
*Oscillatoria*	5	NA	NA	NA	NA	NA	NA	NA	NA	NA	NA	NA	NA
*Phormidum*	1	NA	–	CC	NA	NA	CC	NA	NA	–	NA	NA	–
*Euglena*	5	23S, CC	23S, 16S, CC	23S, 16S, CC	23S	23S	23S, 16S, CC	–	23S	23S	–	23S	23S, CC
*Lepocinclis*	1	–	16S	CC	CC	CC	23S, 16S, CC	–	23S	23S	23S, CC	23S, CC	23S, CC
*Phacus*	2	CC	23S, 16S, CC	–	CC	CC	23S, 16S, CC	–	–	23S	CC	–	23S, CC
*Score*	44	11	26	21	22	16	28	11	17	16	14	13	30
		Low	High	High	High	Moderate	High	Low	Moderate	Moderate	Low	Low	High

Scores were assigned if a genus had an average relative abundance ≥ 0.50% and was present in at least 3 samples for that given year and source for at least one dataset (cell count data or 23S/18S/16S rRNA gene data). “CC” indicates cell count, “NA” indicates complete absence of the genus and “-” indicates the genus is present with < 0.50% average relative abundance. “Total” indicates the score for all years combined. Scores ≥ 20 indicate clear evidence of high organic pollution, those ranging from 15–19 were moderate and were considered to have probable evidence of organic pollution, and scores < 15 were low and were considered to have no evidence of organic pollution [45]

### Quantification of phytoplankton over time

**Fig. 8:**
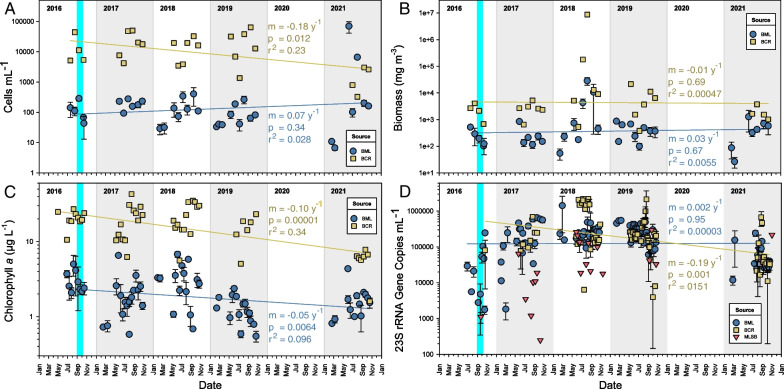
Quantification of phytoplankton for 2016–2021 based on cell counts (**A**), biomass (**B**), chlorophyll *a* (**C**), and qPCR quantification of the 23S rRNA gene (**D**) for surface water samples. Given are the slope (m) per year, p-values based on a t-test of slope = 0, and the r^2^ value for the regression lines. Data points are means of three platforms ± 1 SEM for BML; points for MLSB and BCR are single values. The teal bar indicates alum addition

Quantitative measurements of phytoplankton generally showed higher values in the control site BCR than in BML (Fig. 8). Only the 23S rRNA gene qPCR assay showed comparable or higher populations in BML compared to BCR (Fig. 8D). Counts based on the qPCR assay were 2–4 orders of magnitude higher than microscopic counts, likely due to biases in molecular versus microscopic methods. qPCR can be biased since some organisms carry more than one copy of the 23S rRNA gene, leading to overestimation. Conversely, in the microscopic methods, many morphologically indistinct phytoplankton are overlooked, leading to underestimation. Average gene counts in MLSB were usually about one order of magnitude lower than either BML or BCR (Fig. 8D).

At the *p* = 0.01 level, phytoplankton cell counts and biomass did not detectably change in BML over time, i.e., slopes were not significantly different from zero (Fig. 8). Of the four quantification measures, only chlorophyll *a* content significantly changed in BML over time, and this decline was also observed in BCR (Fig. 8C, Additional file 2: Table S26). Because this decline was evident in both sites, it likely reflects climate or other stochastic factors rather than any particular trend in BML. Furthermore, it is possible that this measurement is complicated by the presence of vanadium, which forms a porphyrin structure similar to that of chlorophyll *a* [49]. Overall, a consistent trend in total phytoplankton over the 5-year study could not be concluded.

## Discussion

### Comparison of gene sequencing and microscopy

BML is being thoroughly sampled in an adaptive management program to investigate the viability of EPLs as a reclamation strategy for oil sands mines. In this study, we examined phytoplankton community composition over multiple years using a combination of high-throughput sequencing (HTS) from three different PCR analyses (23S, 18S, and 16S rRNA genes) and microscopic cell count data, each of which have their advantages and drawbacks. For instance, many phytoplankton such as cyanobacteria and picoeukaryotes cannot be distinguished morphologically [50] without the use of additional methodologies such as flow cytometry or fluorescence microscopy, and thus some studies have observed higher phytoplankton diversity estimates with HTS than microscopy [51, 52]. Some organisms also carry multiple copies of rRNA genes, which can introduce biases in relative abundance measures estimated via HTS [53]. For example, the genome for one strain of *Cryptomonas curvata* has seven copies of the 18S rRNA gene (Joint Genome Institute Sequencing Project ID Gp0211829). HTS introduces further biases depending on the primers, DNA extraction methods, and PCR procedures, and some ASVs cannot be properly assigned a taxonomy due to the incomplete state of reference databases [51, 52, 54]. Various works confirm that molecular and microscopy approaches are complementary and their co-application provides a more complete depiction of the whole phytoplankton community [51]. However, few phytoplankton studies compare microscopy to HTS over multiple years (e.g., [52]), and to our knowledge, no study has used this combination of three rRNA gene analyses over multiple years, each of which also have their own benefits and disadvantages.

The 16S rRNA gene can be used to identify both prokaryotic cyanobacteria and eukaryotic chloroplasts. However, heterotrophic bacteria are typically far more abundant than phytoplankton, and often only a small fraction of phytoplankton sequences are detected within a 16S rRNA gene amplicon [54]. Out of ~ 36,600 total bacterial ASV features observed in all our samples, only 2,409 were identified as belonging to phytoplankton (6.6%) (Additional file 2: Table S6). Sequencing of the 18S rRNA gene is commonly used to investigate general eukaryotic diversity [55, 56] and microalgal diversity [34], but the primers used in our study present some constraints. Foremost, the primers do not effectively amplify for Excavates [57], and therefore detected virtually no *Euglenophyceae*. Sequencing of the 18S rRNA gene is also known to estimate a greater proportion of heterotrophic flagellates compared to microscopy observations, particularly with chrysophyte clades [58]. About 5.3 million reads out of 10.5 million total raw reads were identified as heterotrophic flagellates. These were removed from the 18S dataset in an attempt to obtain exclusively microalgae (including mixotrophs; see Additional file 2: Table S5-6), but some heterotrophic flagellates may have remained after filtration as unclassified ochryophyte OTUs (e.g., unassigned *Chrysophyceae*). In contrast to the 16S and 18S rRNA genes, domain V of the 23S rRNA gene has a mostly exclusive, comprehensive coverage of photosynthetic microbial groups [34]. However, the 23S rRNA gene is limited by the size of the sequence databases; the most comprehensive database is currently μgreen-db with just over 2,300 sequences [34].

Using multiple molecular and microscopic analyses helped us overcome some of the limitations of each analysis in identifying key phytoplankton taxa. The analyses largely agreed, lending support to our conclusions. The molecular analyses allowed some investigations not possible via traditional microscopic examinations, such as the documentation of consistent seasonal patterns, and the observation that different sites favoured different strains (ASVs) of the same genera. There appear to be common niches for phytoplankton in both the freshwater reservoir BCR and the end-pit lake BML, resulting in similar genus-level diversity, but the distinct conditions in each habitat may have selected for distinct species and strains.

### Phytoplankton community composition in BML

Based on alpha diversity (Fig. 7) and beta diversity analyses (Figs. 2, 4, Additional file 2: Table S15), each of the three sites were unique. BML was intermediate to the two controls in its alpha diversity, and generally more comparable to BCR than to MLSB in its community composition. This was in-line with expectations, since BML had superior water quality compared to tailings ponds like MLSB [3, 21], and much better clarity after the addition of alum in 2016 [59]. Compared to BCR, BML is dimictic and had lower phosphorous content (Additional file 2: Table S1), factors that affect phytoplankton composition [60]. BML also contained more contaminants such as NAs and higher salinity compared to BCR, and thus more sensitive phytoplankton taxa may not have survived dispersal from BCR (discussed more below) [19]. Compared to BCR, BML’s community composition was also less stable. As shown in Fig. 3, BML had more ephemeral and fewer persistent phytoplankton taxa over the 5-year study period.

Many of the dominant phytoplankton taxa observed in BML are common in boreal lakes or similar freshwater environments. A study of 75 boreal and temperate lakes and reservoirs in Alberta found a similar community composition in natural and constructed aquatic systems, suggesting that local phytoplankton populations establish rapidly within constructed aquatic systems [60]. Dominant phyla in BML included *Cyanobacteria*, *Cryptophyta*, and *Chlorophyta* (Fig. 1 and Additional file 1: Fig. S2), which are characteristic of natural boreal lakes in Canada [47, 61]. The major genera in BML included *Choricystis*, *Synechococcus*, *Cryptomonas*, and *Euglena. Synechococcus* is a typical prokaryotic picophytoplankton constituent in freshwaters [50] such as the large boreal Lake Balaton [62]. The trebouxiophyte *Choricystis* is ubiquitous to freshwater environments [50], and is frequently found in boreal lakes [58, 62], as are cryptophytes such as *Cryptomonas* [47, 54, 58]. Tropical aquatic environments, in contrast, are typically dominated by *Chlorophyceae*, cyanobacteria, and diatoms, with trebouxiophytes and cryptophytes typically absent or lower in abundance [63–65]. The four major genera in BML: *Choricystis*, *Synechococcus*, *Cryptomonas*, and *Euglena,* have also been detected in oil-impacted environments [66, 67]. Some *Chlorophyta*, *Euglenophyceae*, and *Synechococcales* are tolerant of naphthenic acids (NAs) [20, 66, 68] and have been observed in oil sands tailings ponds (Additional file 1: Figs. S3-4) [21]. Overall, BML’s phytoplankton community composition contains many taxa common to natural boreal lakes and petroleum-impacted environments (Fig. 1).

Notably though, the groups *Chrysophyceae*, *Bacillariophyta*, and *Dinophyta* had lower abundances in BML than expected for a boreal lake (Fig. 1, Additional file 1: Figs. S11–S13). In BML’s freshwater input source, BCR, dinophytes occurred in only 2.1% of samples, possibly limiting their dispersal, but chrysophytes and diatoms are more abundant and persistent in BCR than in BML, suggesting their establishment in BML may have been restricted by water quality. Chrysophytes are known to be highly sensitive to environmental changes [69], exposure to oil contamination [70–72], and turbidity [70]. Freshwater chrysophyte diversity is highest in oligotrophic waters with low salinity (conductivity < 40 µs cm^−1^) and acidic pH (< 7.0) [69, 73]. Thus, many chrysophytes may not survive inoculation from BCR to BML, which has a similar pH but an average conductivity almost 7 times greater than BCR’s (Additional file 2: Table S1). Conversely, some lab studies report that chrysophytes persist in marine oil-contaminated mesocosms [74], oil-contaminated peat bogs [75], or microcosms treated with NAs [76]. Diatoms have also been reported as sensitive to oil contamination [75, 76]. However, various pollution-tolerant diatoms commonly persist in oil-contaminated environments [77], or those containing moderate NA concentrations (> 10 mg L^−1^) [18, 19], such as *Nitzschia* and *Navicula*, which are found in BML, BCR, and MLSB. Besides being very scarce in gene sequencing data, cell count data identified dinophytes in only four BML samples, confirming that their scarcity was not due to methodological biases (Additional file 1: Fig. S13). Not all boreal lakes contain substantial proportions of dinophytes [78], and they are also known to be sensitive to oil contamination [74], so their scarcity in BML is not surprising. However, BML displayed a higher overall level of transitory, ephemeral taxa than BCR (Fig. 3), suggesting that many taxa from the BCR source water are unable to establish in BML.

Phytoplankton can serve as indicators of lake trophic status [46]. Most Alberta boreal lakes are eutrophic to hypereutrophic due to high natural and anthropogenic phosphorous content. Cyanobacteria are often the dominant phytoplankton phylum, regardless of anthropogenic impact [8, 79], and are generally more abundant with increasing eutrophication, with greater amounts of *Dolichospermum* and *Microcystis* expected [46]. BML is classified as oligotrophic to mesotrophic (Additional file 2: Table S1) [79] and does not appear to contain a disproportionately larger cyanobacterial population compared to boreal lakes (Fig. 1). BML’s picophytoplankton community is dominated by *Choricystis* and *Synechococcus* (Fig. 6, Additional file 1: Figs. S7 and S9A), which are found in a wide range of trophic states [50, 58, 62, 80] but have competitive advantages in turbid and eutrophic freshwaters. For instance, accessory pigments in *Synechococcus* strains confer adaptations to low-light [50]. Mixotrophs such as *Cryptomonas* and euglenophytes, abundant in BML (e.g., Figs. 1, 6), are also common to eutrophic waters [46, 81] and are favoured in boreal lakes with reduced light penetration [82], particularly those with lower zooplankton abundance [83].

Many of the major phytoplankton members in BML have been reported as significant food web contributors in other systems. Cryptophytes such as *Cryptomonas* serve as a high quality nutrition source to zooplankton [13, 15], ciliates [16], and bacteria [84]. Picophytoplankton such as *Choricystis* and *Synechococcus* are consumed by ciliates and heterotrophic or mixotrophic nanoflagellates [50], but are low-quality nutritional sources lacking polyunsaturated fatty acids and sterols. However, they can be suitable for consumption by smaller protist grazers, by which they can undergo “trophic upgrading”. For instance, the heterotrophic nanoflagellate *Paraphysomonas* (highly abundant in BML based on 18S rRNA gene sequencing; data not shown) consumes these picophytoplankton and synthesizes lipids de novo, providing improved nutrition to higher trophic levels [15]. Other known grazers of *Synechococcus* include cryptophytes and euglenophytes [85, 86]. Only a handful of *Synechococcus* species have been reported to produce microcystins [87], with no cyanotoxin-producing strains reported in Alberta thus far [17, 88]. Filamentous cyanobacteria such as *Prochlorothrix* and *Planktothrix*, abundant in BML and BCR, respectively (Additional file 1: Figs. S3, S4), are known to contribute to food webs in freshwater lakes [89, 90]. However, they can be difficult for filter-feeding zooplankton and protists to ingest [91]; several species of *Planktothrix* are also known to produce cyanotoxins [87, 88]. BML is notably lower in relative abundance for diatoms, dinophytes, and photosynthetic chrysophytes (Fig. 1), which are important nutritional sources in boreal lakes [13]. Nevertheless, BML contains many of the important phytoplankton groups that are the typical bases for boreal lake food webs.

### Phytoplankton seasonality

Seasonal changes in stratification, temperature, turbidity, light, nutrient distribution, and grazing pressure can mediate temporal changes in phytoplankton [92, 93]. Many genera in BML experienced recurring annual patterns of growth (Additional file 1: Figs. S7–S13, Additional file 2: Table S23). *Cryptomonas* usually peaked in early autumn (Fig. 6, Additional file 1: Fig. S8), similar to observations in Norwegian and Swedish boreal lakes [94, 95]. *Choricystis* peaked in abundance in the spring, from May to June in BML (Fig. 6, Additional file 1: Fig. S7), also consistent with other studies [96, 97]. The euglenophytes *Euglena*, *Phacus*, and *Lepocinclis* peaked in spring from May to June, while *Trachelomonas* peaked in autumn (Fig. 6, Additional file 1: Fig. S10). Less abundant groups like diatoms and dinophytes also showed some annual patterns. Diatoms such as *Asterionella* and *Fragilaria* are known to be favoured by mixing periods [98, 99], and those in Additional file 1: Fig. S12 each showed at least one peak during a mixing period in BML. Consistent with our observations on BML, *Asterionella* and *Fragilaria* have been found to increase during spring in lakes, while *Aulacosiera* and *Cyclotella* are known to increase in autumn or the end of summer [100, 101]. Dinophytes in BML increased in autumn for 2018–2021 (Additional file 1: Fig. S13), consistent with other findings [102, 103]. Cyanobacterial blooms are generally favoured by stratification [104], as well as elevated nutrient concentrations and water temperatures [8]. They are common during the late summer or autumn months in Canadian freshwaters [8, 17, 60, 99] and other temperate regions [105], as was the case for *Synechococcus*, *Aphanizomenon*, *Microcystis*, and *Planktothrix* in BML (Fig. 1, Additional file 1: Fig. S9). Annual patterns in BML therefore suggest ecosystem dynamics comparable to those found in natural boreal lakes.

### Phytoplankton quantification

It was predicted that phytoplankton populations would increase over time due to increased water clarity resulting from the 2016 alum addition [7], however, different measurements led to inconsistent conclusions. Chlorophyll *a* measures significantly declined in BML from 2016 to 2021. Conversely, phytoplankton cell count and biomass did not change significantly from 2016 to 2021, while 23S rRNA gene counts appeared to first increase from 2016 to 2019, and then decrease again in 2021, with no net overall linear trend. We are unable to assess whether the addition of alum in 2016 had a major immediate effect on algal growth because of a shortage of pre-2016 samples, but the algal load remained quite stable in the 5 years after clarification.

Phytoplankton gene counts in BML were usually equal or higher than in BCR, but non-molecular measures suggested that phytoplankton were usually one to two orders of magnitude lower in BML than BCR (Fig. 8, Additional file 2: Table S26). BCR had greater nutrient content and therefore likely supported greater phytoplankton abundances. However, some measures indicated a decline in phytoplankton quantity for both sites in 2021 (Fig. 8, Additional file 2: Table S26). Phosphorus, nitrogen, turbidity, and total organic carbon were all lower in 2021 compared to previous years for both sites, while total hardness was about twice as high for BCR (from ~ 125 mg L^−1^ to ~ 230 mg L^−1^) (Additional file 2: Table S1). These changes in BML and BCR in 2021 may have resulted from the pandemic shutdown of mine operations in 2020, including disruption of normal water management. During 2021, no water was pumped in to BML or BCR. More data will be needed to determine long-term trends, and whether 2021 was atypical.

BML and BCR’s phytoplankton biomass and chlorophyll *a* content were within the ranges expected of boreal freshwater lakes. For instance, non-impacted large oligo-humic Finnish boreal lakes had total phytoplankton biomasses and chlorophyll *a* concentrations ranging from 120 to 980 mg m^−3^ and 1.2–8.2 μg L^−1^, respectively, which match closely with ranges in BML (mean ranges were 109–11,005 mg m^−3^ and 1.3–3.4 μg L^−1^; see Additional file 2: Table S26) [106]. BML’s chlorophyll *a* content was also slightly greater than that of post-logging boreal lakes in Ontario, which ranged from 0.9 to 1.2 μg L^−1^ [61]. BCR’s biomass and chlorophyll *a* content were about an order of magnitude higher than BML’s (mean ranges were 1,990–1,499,159 mg m^−3^ and 5.8–21.7 μg L^−1^, respectively; see Additional file 2: Table S26), and compared more closely to wetland-dominated Alberta boreal lakes, which had mean biomass and chlorophyll *a* concentrations of ~ 6,400 mg m^−3^ and 21 μg L^−1^, respectively [47].

### Phytoplankton diversity over time

The relative abundances of *Cryptophyta*, *Chlorophyta*, and *Cyanobacteria* did not change substantially over 5 years in BML (Figs. 1, 6, and Additional file 1: Figs. S7-S9). However, changes in some other taxa may indicate a changing system. Haptophytes and dinophytes increased in later years in BML (Additional file 1: Fig. S13), while remaining unchanged in the BCR control site. Dinophytes are natural constituents of boreal lakes (e.g., [47]), while haptophytes are predominantly marine organisms [107], but have also been found in freshwaters in small quantities [61, 108]. Despite the slightly saline conditions in BML, the haptophytes observed were freshwater genera belonging to *Pavlovales*. The increase in these taxa suggests that new species may be colonizing BML as conditions improve. In contrast, many ochrophytes and diatoms, after appearing to increase in 2018–2019, were lowest in abundance during 2021 (Additional file 1: Figs. S11-S12). As noted above, shutdown of pump-in to BML from BCR in 2021 may be responsible for some of these changes, although many taxa still persisted in BML (e.g., Fig. 6, Additional file 1: Fig. S5).

New aquatic systems may become more biologically diverse over time as reclamation proceeds and new species establish [109]. BML evidently represents an intermediate state between a tailings pond and a freshwater reservoir, both in its alpha diversity and beta diversity (Fig. 7, Additional file 1: Figs. S14-S15). The more biodiverse freshwater reservoir BCR is a source of phytoplankton inoculation into BML, and diversity is expected to increase over time in BML relative to BCR as water quality improves. However, phytoplankton α-diversity did not consistently increase or decrease in BML over time based on different diversity metrics and gene sequencing datasets (Additional file 1: Figs. S14–S16 and Additional file 2: Table S25). Any positive trends were small, inconsistent, and explained little of the overall variation. Long-term trends may have been masked by year-to-year and seasonal variability. BML’s phytoplankton α-diversity experienced increases during autumn turnover (Additional file 1: Fig. S15), whereas BCR showed no clear seasonal patterns, a difference likely due to the lack of turnover periods and/or insufficient sampling of BCR. Disturbances such as the alum addition and nearby wildfires in 2016 also could have contributed to the observed variability. A study on boreal plain lakes in Alberta concluded that wildfires impacted phytoplankton communities in those lakes for 4 years after the fire [110]. More data will be necessary to conclude whether there are long-term trends in BML α-diversity.

## Conclusion

This research establishes a baseline of phytoplankton community composition in an oil sands end pit lake based on both DNA sequencing and cell count methods. These data can be used to inform planning and management of future oil sands end pit lakes [7]. We have demonstrated the presence of phytoplankton taxa in BML comparable to those found in natural boreal lakes, with potential food web members present. BML shows distinct seasonal patterns in some phytoplankton taxa consistent with natural boreal lakes. Neither phytoplankton abundance nor alpha diversity increased notably in 5 years from 2016 to 2021, but data from future years could resolve this. BML diversity was intermediate between the freshwater and tailings controls. BML had a community composition similar to the freshwater control at higher taxonomic levels but differences were evident at the strain level, with fewer persistent strains in BML. Phytoplankton abundance and seasonality in BML were not merely a product of freshwater inflow. Instead, specific phytoplankton strains established and continued to exhibit seasonal patterns without freshwater input in 2021. Further research should continue to monitor the phytoplankton community in BML in terms of its community composition, seasonality, and diversity over time. Additional research could also clarify the roles of different phytoplankton groups in the BML food web.

### Supplementary Information

**Additional file 1**. Notes and figures.

**Additional file 2**. Tables.

## Data Availability

Amplicon sequencing data is publicly available in the SRA repository under Accession no. PRJNA1003951.

## Abbreviations

BCR: Beaver Creek Reservoir
BML: Base Mine Lake
MLSB: Mildred Lake Settling Basin

## References

[1] Government of Alberta. Alberta Oilsands Facts and Statistics. Alberta. 2023. https://www.alberta.ca/oil-sands-facts-and-statistics 2023. Accessed 2 December 2023.

[2] Kannel PR, Gan TY (2012). Naphthenic acids degradation and toxicity mitigation in tailings wastewater systems and aquatic environments: a review. J Environ Sci Health Part A.

[3] Foght JM, Gieg LM, Siddique T (2017). The microbiology of oil sands tailings: past, present, future. FEMS Microbiol Ecol.

[4] Alberta Energy Regulator (AER). State of Fluid Tailings Management for Mineable Oil Sands, 2020. Alberta Energy Regulator. 2021. https://static.aer.ca/prd/documents/reports/2020-State-Fluid-Tailings-Management-Mineable-OilSands.pdf. Accessed 2 December 2023.

[5] Richardson E, Dacks JB (2019). Microbial Eukaryotes in Oil Sands Environments: Heterotrophs in the Spotlight. Microorganisms.

[6] Cossey HL, Batycky AE, Kaminsky H, Ulrich AC (2021). Geochemical Stability of Oil Sands Tailings in Mine Closure Landforms. Minerals.

[7] Syncrude Canada Ltd. 2022 Pit Lake Monitoring and Research Report (Base Mine Lake Demonstration Summary: 2012–2021). Syncrude Canada Ltd. 2022. https://era.library.ualberta.ca/items/0994fedb-74d0-4397-a6ad-932f78bee1ee. Accessed 2 December 2023.

[8] Cumulative Environmental Management Association (CEMA). End Pit Lake Guidance Document 2012. Cumulative Environmental Management Association. 2012. https://www.cclmportal.ca/sites/default/files/2022-01/CEMA%20EPL%20Guide.pdf. Accessed 2 December 2023.

[9] Kabwe LK, Scott JD, Beier NA, Wilson GW, Jeeravipoolvarn S (2019). Environmental implications of end pit lakes at oil sand mines in Alberta. Canada Environmental Geotechnics.

[10] Gammons C, Harris L, Castro J, Cott P, Hanna B. Creating Lakes from Open Pit Mines: Processes and Considerations, Emphasis on Northern Environments. 2009.

[11] Risacher FF, Morris PK, Arriaga D, Goad C, Nelson TC, Slater GF (2018). The interplay of methane and ammonia as key oxygen consuming constituents in early stage development of Base Mine Lake, the first demonstration oil sands pit lake. Appl Geochem.

[12] Pal R, Choudhury AK, Pal R, Choudhury AK (2014). A brief introduction to phytoplanktons. An introduction to phytoplanktons.

[13] Galloway AWE, Taipale SJ, Hiltunen M, Peltomaa E, Strandberg U, Brett MT (2014). Diet-specific biomarkers show that high-quality phytoplankton fuels herbivorous zooplankton in large boreal lakes. Freshw Biol.

[14] Strandberg U, Hiltunen M, Jelkänen E, Taipale SJ, Kainz MJ, Brett MT (2015). Selective transfer of polyunsaturated fatty acids from phytoplankton to planktivorous fish in large boreal lakes. Sci Total Environ.

[15] Bec A, Martin-Creuzburg D, von Elert E (2006). Trophic upgrading of autotrophic picoplankton by the heterotrophic nanoflagellate *Paraphysomonas* sp. Limnol Oceanogr.

[16] Flöder S, Yong J, Klauschies T, Gaedke U, Poprick T, Brinkhoff T (2021). Intraspecific trait variation alters the outcome of competition in freshwater ciliates. Ecol Evol.

[17] Kotak BG, Zurawell RW (2007). Cyanobacterial toxins in Canadian freshwaters: a review. Lake Reservoir Manag.

[18] Leung SS-C, MacKinnon MD, Smith REH (2001). Aquatic reclamation in the Athabasca, Canada, oil sands: Naphthenate and salt effects on phytoplankton communities. Environ Toxicol Chem.

[19] Leung SS, MacKinnon MD, Smith REH (2003). The ecological effects of naphthenic acids and salts on phytoplankton from the Athabasca oil sands region. Aquat Toxicol.

[20] Quagraine EK, Peterson HG, Headley JV (2005). In situ bioremediation of naphthenic acids contaminated tailing pond waters in the Athabasca Oil Sands Region—demonstrated field studies and plausible options: a review. J Environ Sci Health Part A.

[21] Aguilar M, Richardson E, Tan B, Walker G, Dunfield PF, Bass D (2016). Next-generation sequencing assessment of eukaryotic diversity in oil sands tailings ponds sediments and surface water. J Eukaryot Microbiol.

[22] Richardson E, Bass D, Smirnova A, Paoli L, Dunfield P, Dacks JB (2020). Phylogenetic estimation of community composition and novel eukaryotic lineages in Base Mine Lake: an oil sands tailings reclamation site in Northern Alberta. J Eukaryot Microbiol.

[23] Albakistani EA, Nwosu FC, Furgason C, Haupt ES, Smirnova AV, Verbeke TJ (2022). Seasonal dynamics of methanotrophic bacteria in a boreal oil sands end pit lake. Appl Environ Microbiol.

[24] Corkum L, Thompson MV, Hamilton HR (1985). Water quality overview of Athabasca River Basin. Univ Alberta Libr.

[25] Rachel NM, Gieg LM (2020). Preserving microbial community integrity in oilfield produced water. Front Microbiol.

[26] Ladell BA, Walleser LR, McCalla SG, Erickson RA, Amberg JJ (2019). Ethanol and sodium acetate as a preservation method to delay degradation of environmental DNA. Conserv Genet Resour.

[27] Sherwood AR, Presting GG (2007). Universal primers amplify a 23S rDNA plastid marker in eukaryotic algae and cyanobacteria. J Phycol.

[28] Stoeck T, Bass D, Nebel M, Christen R, Jones MDM, Breiner H-W (2010). Multiple marker parallel tag environmental DNA sequencing reveals a highly complex eukaryotic community in marine anoxic water. Mol Ecol.

[29] Klindworth A, Pruesse E, Schweer T, Peplies J, Quast C, Horn M (2013). Evaluation of general 16S ribosomal RNA gene PCR primers for classical and next-generation sequencing-based diversity studies. Nucleic Acids Res.

[30] Caporaso JG, Kuczynski J, Stombaugh J, Bittinger K, Bushman FD, Costello EK (2010). QIIME allows analysis of high-throughput community sequencing data. Nat Methods.

[31] Martin M (2011). Cutadapt removes adapter sequences from high-throughput reads. EMBnet J..

[32] Callahan BJ, McMurdie PJ, Rosen MJ, Han AW, Johnson AJA, Holmes SP (2016). DADA2: High-resolution sample inference from Illumina amplicon data. Nat Methods.

[33] Bokulich NA, Kaehler BD, Rideout JR, Dillon M, Bolyen E, Knight R (2018). Optimizing taxonomic classification of marker-gene amplicon sequences with QIIME 2’s q2-feature-classifier plugin. Microbiome.

[34] Djemiel C, Plassard D, Terrat S, Crouzet O, Sauze J, Mondy S (2020). µgreen-db: a reference database for the 23S rRNA gene of eukaryotic plastids and cyanobacteria. Sci Rep.

[35] Quast C, Pruesse E, Yilmaz P, Gerken J, Schweer T, Yarza P (2013). The SILVA ribosomal RNA gene database project: improved data processing and web-based tools. Nucleic Acids Res.

[36] Pruesse E, Peplies J, Glöckner FO (2012). SINA: Accurate high-throughput multiple sequence alignment of ribosomal RNA genes. Bioinformatics.

[37] Altschul S, Gish W, Miller W, Myers E, Lipman D (1990). Basic local alignment search tool. J Mol Biol.

[38] Hillebrand H, Dürselen C-D, Kirschtel D, Pollingher U, Zohary T (1999). Biovolume calculation for pelagic and benthic microalgae. J Phycol.

[39] Welschmeyer NA (1994). Fluorometric analysis of chlorophyll a in the presence of chlorophyll b and pheopigments. Limnol Oceanogr.

[40] Dixon P (2003). VEGAN, a package of R functions for community ecology. J Veg Sci.

[41] Beule L, Karlovsky P (2020). Improved normalization of species count data in ecology by scaling with ranked subsampling (SRS): application to microbial communities. PeerJ.

[42] Somerfield PJ, Clarke KR, Gorley RN (2021). Analysis of similarities (ANOSIM) for 2-way layouts using a generalised ANOSIM statistic, with comparative notes on Permutational Multivariate Analysis of Variance (PERMANOVA). Austral Ecol.

[43] De Cáceres M, Legendre P, Wiser SK, Brotons L (2012). Using species combinations in indicator value analyses. Methods Ecol Evol.

[44] Benjamini Y, Hochberg Y (1995). Controlling the false discovery rate: a practical and powerful approach to multiple testing. J Roy Stat Soc B.

[45] Palmer CM (1969). A composite rating of algae tolerating organic pollution. J Phycol.

[46] Bellinger EG, Sigee DC (2015). Freshwater algae: identification, enumeration and use as bioindicators.

[47] Prepas EE, Vitt DH, Dinsmore WP, Halsey LA, McEachern PM, Scrimgeour GJ (2001). Landscape variables influencing nutrients and phytoplankton communities in Boreal Plain lakes of northern Alberta: a comparison of wetland- and upland-dominated catchments. Can J Fish Aquat Sci.

[48] Chow C-ET, Sachdeva R, Cram JA, Steele JA, Needham DM, Patel A (2013). Temporal variability and coherence of euphotic zone bacterial communities over a decade in the Southern California Bight. ISME J.

[49] Dechaine GP, Gray MR (2010). Chemistry and association of vanadium compounds in heavy oil and bitumen, and implications for their selective removal. Energy Fuels.

[50] Callieri C (2007). Picophytoplankton in freshwater ecosystems: the importance of small-sized phototrophs. Freshwater Rev.

[51] Vuorio K, Mäki A, Salmi P, Aalto SL, Tiirola M (2020). Consistency of targeted metatranscriptomics and morphological characterization of phytoplankton communities. Front Microbiol.

[52] Obertegger U, Pindo M, Flaim G (2020). Do inferences about freshwater phytoplankton communities change when based on microscopy or high-throughput sequencing data?. Freshw Biol.

[53] Gong W, Marchetti A (2019). Estimation of 18S gene copy number in marine eukaryotic plankton using a next-generation sequencing approach. Front Mar Sci.

[54] Eiler A, Drakare S, Bertilsson S, Pernthaler J, Peura S, Rofner C (2013). Unveiling distribution patterns of freshwater phytoplankton by a next generation sequencing based approach. PLoS ONE.

[55] Hu SK, Liu Z, Lie AAY, Countway PD, Kim DY, Jones AC (2015). Estimating protistan diversity using high-throughput sequencing. J Eukaryot Microbiol.

[56] Choi J, Park JS (2020). Comparative analyses of the V4 and V9 regions of 18S rDNA for the extant eukaryotic community using the Illumina platform. Sci Rep.

[57] del Campo J, Pons MJ, Herranz M, Wakeman KC, del Valle J, Vermeij MJA (2019). Validation of a universal set of primers to study animal-associated microeukaryotic communities. Environ Microbiol.

[58] Luo W, Bock C, Li HR, Padisák J, Krienitz L (2011). Molecular and microscopic diversity of planktonic eukaryotes in the oligotrophic Lake Stechlin (Germany). Hydrobiologia.

[59] Tedford E, Halferdahl G, Pieters R, Lawrence GA (2019). Temporal variations in turbidity in an oil sands pit lake. Environ Fluid Mech.

[60] Loewen CJG, Wyatt FR, Mortimer CA, Vinebrooke RD, Zurawell RW (2020). Multiscale drivers of phytoplankton communities in north-temperate lakes. Ecol Appl.

[61] Nicholls KH, Steedman RJ, Carney EC (2003). Changes in phytoplankton communities following logging in the drainage basins of three boreal forest lakes in northwestern Ontario (Canada), 1991–2000. Can J Fish Aquat Sci.

[62] Somogyi B, Felföldi T, Tóth LG, Bernát G, Vörös L (2020). Photoautotrophic picoplankton: a review on their occurrence, role and diversity in Lake Balaton. Biol Futura.

[63] Ndebele-Murisa MR, Musil CF, Raitt L (2010). A review of phytoplankton dynamics in tropical African lakes. S Afr J Sci.

[64] Machado KB, Teresa FB, Vieira LCG, Huszar VLDM, Nabout JC (2016). Comparing the effects of landscape and local environmental variables on taxonomic and functional composition of phytoplankton communities. J Plankton Res.

[65] Rahman MdS, Ahmed MF, Islam MS, Moniruzzaman M (2022). A review of the physicochemical features and phytoplankton community of the Bay of Bengal: Bangladesh perspective. Authorea.

[66] Ruffell SE, Frank RA, Woodworth AP, Bragg LM, Bauer AE, Deeth LE (2016). Assessing the bioremediation potential of algal species indigenous to oil sands process-affected waters on mixtures of oil sands acid extractable organics. Ecotoxicol Environ Saf.

[67] Jafari N, Gunale V (2006). Hydrobiological study of algae of an urban freshwater river. J Appl Sci Environ Manag.

[68] Woodworth APJ, Frank RA, McConkey BJ, Müller KM (2012). Toxic effects of oil sand naphthenic acids on the biomass accumulation of 21 potential phytoplankton remediation candidates. Ecotoxicol Environ Saf.

[69] Siver PA, Lott AM (2017). The scaled chrysophyte flora in freshwater ponds and lakes from Newfoundland, Canada, and their relationship to environmental variables. Cryptogamie, Algologie.

[70] Bessudova A, Gabyshev V, Firsova A, Gabysheva O, Bukin Y, Likhoshway Y (2021). Diversity variation of silica-scaled Chrysophytes related to differences in physicochemical variables in estuaries of rivers in an Arctic watershed. Sustainability.

[71] Cederwall J, Black TA, Blais JM, Hanson ML, Hollebone BP, Palace VP (2020). Life under an oil slick: response of a freshwater food web to simulated spills of diluted bitumen in field mesocosms. Can J Fish Aquat Sci.

[72] Lengyel E, Barreto S, Padisák J, Stenger-Kovács C, Lázár D, Buczkó K (2023). Contribution of silica-scaled chrysophytes to ecosystems services: a review. Hydrobiologia.

[73] Němcová Y, Pusztai M, Škaloudová M, Neustupa J (2016). Silica-scaled chrysophytes (Stramenopiles, Ochrophyta) along a salinity gradient: a case study from the Gulf of Bothnia western shore (northern Europe). Hydrobiologia.

[74] Finkel ZV, Liang Y, Nanjappa D, Bretherton L, Brown CM, Quigg A (2020). A ribosomal sequence-based oil sensitivity index for phytoplankton groups. Mar Pollut Bull.

[75] Skorobogatova O, Yumagulova E, Storchak T, Barinova S (2019). Bioindication of the influence of oil production on sphagnum bogs in the Khanty-Mansiysk autonomous Okrug-Yugra, Russia. Diversity.

[76] Hayes TME (2006). Examining the ecological effects of naphthenic acids and major ions on phytoplankton in the Athabasca oil sands.

[77] Faria DMD, Costin JC, Tremarin PI, Ludwig TAV (2019). Temporal changes in biological traits of diatom communities in response to an oil spill in a subtropical river. An Acad Bras Ciênc.

[78] Vogt RJ, St-Gelais NF, Bogard MJ, Beisner BE, del Giorgio PA (2017). Surface water CO_2_ concentration influences phytoplankton production but not community composition across boreal lakes. Ecol Lett.

[79] Alberta Environment and Parks: Trophic State of Alberta Lakes. http://environment.alberta.ca/apps/EdwReportViewer/TrophicStateAlbertaLakes.aspx (2023). Accessed 2 December 2023.

[80] Callieri C, Coci M, Corno G, Macek M, Modenutti B, Balseiro E (2013). Phylogenetic diversity of nonmarine picocyanobacteria. FEMS Microbiol Ecol.

[81] Lepistö L, Rosenström U. The most typical phytoplankton taxa in four types of boreal lakes. In Alvarez-Cobelas M, Reynolds CS, Sánchez-Castillo P, Kristiansen J (eds): Phytoplankton and Trophic Gradients, Dordrecht, Springer Netherlands: 1998:89–97. 10.1007/978-94-017-2668-9_7.

[82] Cantin A, Beisner BE, Gunn JM, Prairie YT, Winter JG (2011). Effects of thermocline deepening on lake plankton communities. Can J Fish Aquat Sci.

[83] Hansson TH, Grossart H, Giorgio PA, St-Gelais NF, Beisner BE (2019). Environmental drivers of mixotrophs in boreal lakes. Limnol Oceanogr.

[84] Šimek K, Kasalický V, Zapomělová E, Horňák K (2011). Alga-derived substrates select for distinct betaproteobacterial lineages and contribute to niche separation in *Limnohabitans strains*. Appl Environ Microbiol.

[85] Yoo YD, Seong KA, Jeong HJ, Yih W, Rho J-R, Nam SW (2017). Mixotrophy in the marine red-tide cryptophyte *Teleaulax amphioxeia* and ingestion and grazing impact of cryptophytes on natural populations of bacteria in Korean coastal waters. Harmful Algae.

[86] Yoo YD, Seong KA, Kim HS, Jeong HJ, Yoon EY, Park J (2018). Feeding and grazing impact by the bloom-forming euglenophyte *Eutreptiella eupharyngea* on marine eubacteria and cyanobacteria. Harmful Algae.

[87] Bernard C, Ballot A, Thomazeau S, Maloufi S, Furey A, Mankiewicz-Boczek J, et al. Appendix 2: Cyanobacteria associated with the production of cyanotoxins. 1st ed. In: Meriluoto J, Spoof L, Codd GA, editors. Handbook of Cyanobacterial Monitoring and Cyanotoxin Analysis. West Sussex: John Wiley & Sons Ltd: 2017. p. 1–25.

[88] Government of Canada. Cyanobacterial toxins in drinking water. In: Consultation on cyanobacterial toxins in drinking water. Health Canada. 2016. https://www.canada.ca/en/health-canada/programs/cyanobacterial-toxins-drinking-water/cyanobacterial-toxins-drinking-water.html. Accessed 2 December 2023.

[89] Perga M-E, Domaizon I, Guillard J, Hamelet V, Anneville O (2013). Are cyanobacterial blooms trophic dead ends?. Oecologia.

[90] Gulati RD, Ejsmont-Karabin J (1993). Feeding in *Euchlanis dilatata lucksiana* Hauer on filamentous cyanobacteria and a prochlorophyte. Hydrobiologia.

[91] Dirren S, Pitsch G, Silva MOD, Posch T (2017). Grazing of Nuclearia thermophila and Nuclearia delicatula (Nucleariidae, Opisthokonta) on the toxic cyanobacterium Planktothrix rubescens. Eur J Protistol.

[92] Reynolds CS, Huszar V, Kruk C, Naselli-Flores L, Melo S (2002). Towards a functional classification of the freshwater phytoplankton. J Plankton Res.

[93] Berger SA, Diehl S, Stibor H, Trommer G, Ruhenstroth M, Wild A (2006). Water temperature and mixing depth affect timing and magnitude of events during spring succession of the plankton. Oecologia.

[94] Brettum P, Halvorsen G (2004). The phytoplankton of Lake Atnsjøen, Norway: a long-term investigation. Hydrobiologia.

[95] Ramberg L (1979). Relations between phytoplankton and light climate in two Swedish forest lakes. Internationale Revue der gesamten Hydrobiologie und Hydrographie.

[96] Hepperle D, Krienitz L (2001). Systematics and ecology of Chlorophyte picoplankton in German inland waters along a nutrient gradient. Int Rev Hydrobiol.

[97] Hepperle D, Schlegel I (2002). Molecular diversity of eucaryotic picoalgae from three lakes in Switzerland. Int Rev Hydrobiol.

[98] Reynolds CS, Wiseman SW, Godfrey BM, Butterwick C (1983). Some effects of artificial mixing on the dynamics of phytoplankton populations in large limnetic enclosures. J Plankton Res.

[99] Loewen CJG, Vinebrooke RD, Zurawell RW (2021). Quantifying seasonal succession of phytoplankton trait-environment associations in human-altered landscapes. Limnol Oceanogr.

[100] Tsukada H, Tsujimura S, Nakahara H (2006). Seasonal succession of phytoplankton in Lake Yogo over 2 years: effect of artificial manipulation. Limnology.

[101] Carrillo P, Reche I, Sanchez-Castillo P, Cruz-Pizarro L (1995). Direct and indirect effects of grazing on the phytoplankton seasonal succession in an oligotrophic lake. J Plankton Res.

[102] Keskitalo J, Salonen K, Holopainen A-L (1998). Long-term fluctuations in environmental conditions, plankton and macrophytes in a humic lake. Valkea-Kotinen Boreal Environ Res.

[103] Carrick HJ (2005). An under-appreciated component of biodiversity in plankton communities: the role of Protozoa in Lake Michigan (a case study). Hydrobiologia.

[104] Visser PM, Ibelings BW, Bormans M, Huisman J (2016). Artificial mixing to control cyanobacterial blooms: a review. Aquat Ecol.

[105] E & FN Spon. Toxic cyanobacteria in water: a guide to their public health consequences, monitoring, and management. London; New York: 1999.

[106] Lepistö L, Holopainen A-L, Vuoristo H (2004). Type-specific and indicator taxa of phytoplankton as a quality criterion for assessing the ecological status of Finnish boreal lakes. Limnologica.

[107] Simon M, López-García P, Moreira D, Jardillier L (2013). New haptophyte lineages and multiple independent colonizations of freshwater ecosystems: new insights into haptophyte diversity. Environ Microbiol Rep.

[108] Richards TA, Vepritskiy AA, Gouliamova DE, Nierzwicki-Bauer SA (2005). The molecular diversity of freshwater picoeukaryotes from an oligotrophic lake reveals diverse, distinctive and globally dispersed lineages. Environ Microbiol.

[109] Stage Sø J, Sand-Jensen K, Baastrup-Spohr L (2020). Temporal development of biodiversity of macrophytes in newly established lakes. Freshw Biol.

[110] Charette T, Prepas EE (2003). Wildfire impacts on phytoplankton communities of three small lakes on the Boreal Plain, Alberta, Canada: a paleolimnological study. Can J Fish Aquat Sci.

