# Supplementary material for: Phytoplankton ecology in the early years of a boreal oil sands end pit lake
Source: Environ Microbiome. 2024 Jan 12;19:3. doi: 10.1186/s40793-023-00544-3 (PMC10787447; doi:10.1186/s40793-023-00544-3)
Supplement: Supplementary file 1 — Additional file 1. Notes and figures. [file 40793_2023_544_MOESM1_ESM.docx]

**Additional File 1: Supplemental Notes and Figures**

**Phytoplankton ecology in the early years of a boreal oil sands end-pit lake**

Chantel C. Furgason^1^, Angela V. Smirnova^1^, Joel B. Dacks^2^, Peter F. Dunfield^1*^

^1^ Department of Biological Sciences, University of Calgary, 2500 University Dr. NW Calgary AB, Canada

^2^ Department of Medicine, University of Alberta, 116 St. and 85 Ave., Edmonton, AB, Canada

*Correspondence should be addressed to: P.F.D. (Email: pfdunfie@ucalgary.ca) and C.C.F (Email: chantel.furgason@ucalgary.ca)

**Supplementary Note 1. *Taxonomic Reassignments.***

ASVs originally identified by SILVA as *Picochlorum* or *Choricystis* for the 18S rRNA gene were reassigned based on observations of relative abundance patterns over time and BLAST closest matches [1].

ASVs originally assigned by SILVA as *Picochlorum* for the 18S rRNA gene had relative abundance patterns comparable to those ASVs identified as *Choricystis* by the 23S rRNA gene (Figs. 6 and S5). The same 18S rRNA gene *Picochlorum* ASVs had BLAST closest matches with *Choricystis* or *Nannochloris atomus* SAG 14.87 (>99%), which is phylogenetically affiliated with *Choricystis* [3,4]. Conversely, ASVs originally assigned as *Choricystis* for the 18S rRNA gene had lower relative abundances over time and no patterns matching the 23S rRNA gene data for *Choricystis* or 18S rRNA gene data for *Picochlorum*. Instead, the 18S rRNA gene ASVs originally assigned as *Choricystis* matched the relative abundance patterns found with *Picochlorum* OTU identified in the 16S rRNA gene dataset (Fig. S5). For these reasons, the names for *Choricystis* and *Picochlorum* were swapped in the 18S rRNA gene dataset and treated as separate OTUs in this work (Table S11).

Similarly, ASVs identified by AlgaeBase as *Cyanobium* and *Synechococcus* for the 23S rRNA gene were merged into a single OTU because combined they had comparable relative abundance patterns with those identified as *Cyanobium* by the SILVA database for the 16S rRNA gene (Fig. 6). *Cyanobium* and *Synechococcus* are heterotypic synonyms (NCBI) and no taxonomic consensus has been reached for distinguishing each [5]. For these reasons, the OTUs identified as *Synechococcus* and *Cyanobium* in the 23S rRNA gene dataset were merged and the name *Synechococcus* is used to indicate both *Cyanobium* and *Synechococcus* sequences in this paper (Table S11).

**Supplemental Note 2. *List of phytoplankton genera counted by microscopy data.***

*Bacillariophyta*: *Achnanthidium*, *Amphora*, *Asterionella*, *Aulacoseira*, *Campylodiscus*, *Cocconeis*, *Cyclotella*, *Cymbella*, *Cymbopleura*, *Diatoma*, *Encyonema*, *Entomoneis, Epithemia*, *Eunotia*, *Fragilaria*, *Gomphonema*, *Gyrosigma*, *Navicula*, *Nitzschia*, *Pseudostaurosira*, *Rhizosolenia*, *Rhoicosphenia*, *Rhopalodia*, *Stephanodiscus*, *Synedra*, *Tabellaria.*

*Chlorophyta: Actinastrum*, *Acutodesmus*, *Ankistrodesmus*, *Botryococcus*, *Carteria*, *Chlamydomonas*, *Chlorella*, *Closteriopsis*, *Closterium*, *Coelastrum*, *Cosmarium*, *Crucigenia*, *Crucigeniella*, *Desmodesmus*, *Dictyosphaerium*, *Elakatothrix*, *Geminella*, *Gonium*, *Kirchneriella*, *Lagerheimia*, *Micractinium*, *Monoraphidium*, *Mougeotia*, *Oedogonium*, *Oocystis*, *Pandorina*, *Pediastrum*, *Quadrigula*, *Roya*, *Scenedesmus*, *Schroederia*, *Selenastrum*, *Sorastrum*, *Spirogyra*, *Staurastrum*, *Stauridium*, *Tetraedron*, *Tetrastrum*, *Ulothrix.*

*Chrysophyceae*: *Chrysococcus*, *Dinobryon*, *Mallomonas*, *Ochromonas*, *Pseudokephyrion*, *Synura.*

*Cryptophyta*: *Chroomonas*, *Cryptomonas*, *Komma*, *Plagioselmis.*

*Cyanobacteria*: *Anabaena*, *Aphanizomenon*, *Aphanocapsa*, *Aphanothece*, *Chroococcus, Heteroleibleinia*, *Leptolyngbya*, *Merismopedia*, *Microcystis*, *Phormidium*, *Planktolyngbya*, *Pseudanabaena*, *Snowella*, *and Woronichinia.*

*Dictyophyceae*: *Pseudopedinella.*

*Dinophyceae*: *Amphidinium*, *Ceratium*, *Gymnodinium*, *Peridinium*.

*Euglenophyceae*: *Euglena*, *Lepocinclis*, *Monomorphina*, *Phacus*, *Strombomonas*, *Trachelomonas*.

*Xanthophyceae*: *Goniochloris*, *Isthmochloron*, *Ophiocytium*, *Tetraëdriella.*

**Supplemental Note 3. *Replacement of Choricystis in 2021.***

Time-course plots (Fig. S5) and species indicator results (Fig. 5, Table S22) suggest *Choricystis* has been replaced in 2021 by *Mychonastes* and unassigned Chlorophyta based on 23S rRNA gene data, and unassigned Trebouxiophyceae based on 18S rRNA gene data. A single unassigned Chlorophyta ASV was found in the 23S rRNA dataset with high reads for BML in 2021 (53,638 reads versus 1,084 reads total for prior years). A BLAST search with this ASV revealed close matches (100% query cover, >97% identity) to various genera from Viridiplantae, including *Geminella minor*, *Interfilum terricola*, and *Oocystis apiculate*. For the 18S rRNA gene, a single unassigned Trebouxiophyceae ASV was also found with high read quantities for BML in 2021 (140,471 reads compared to 47 reads total in prior years). A BLAST search with this sequence showed the closest matches (100% query cover, >99% identity) were species from the order Chlorellales including *Oocystaceae* sp*.*, several species of *Oocystis*, *Oocystella heteromucosa*, and *Chlorella* sp. These results suggest *Choricystis* has been ecologically replaced in BML as of 2021 by *Mychonastes* and an uncultured Chlorophyte related to *Oocystis*.

**Supplementary Figures**


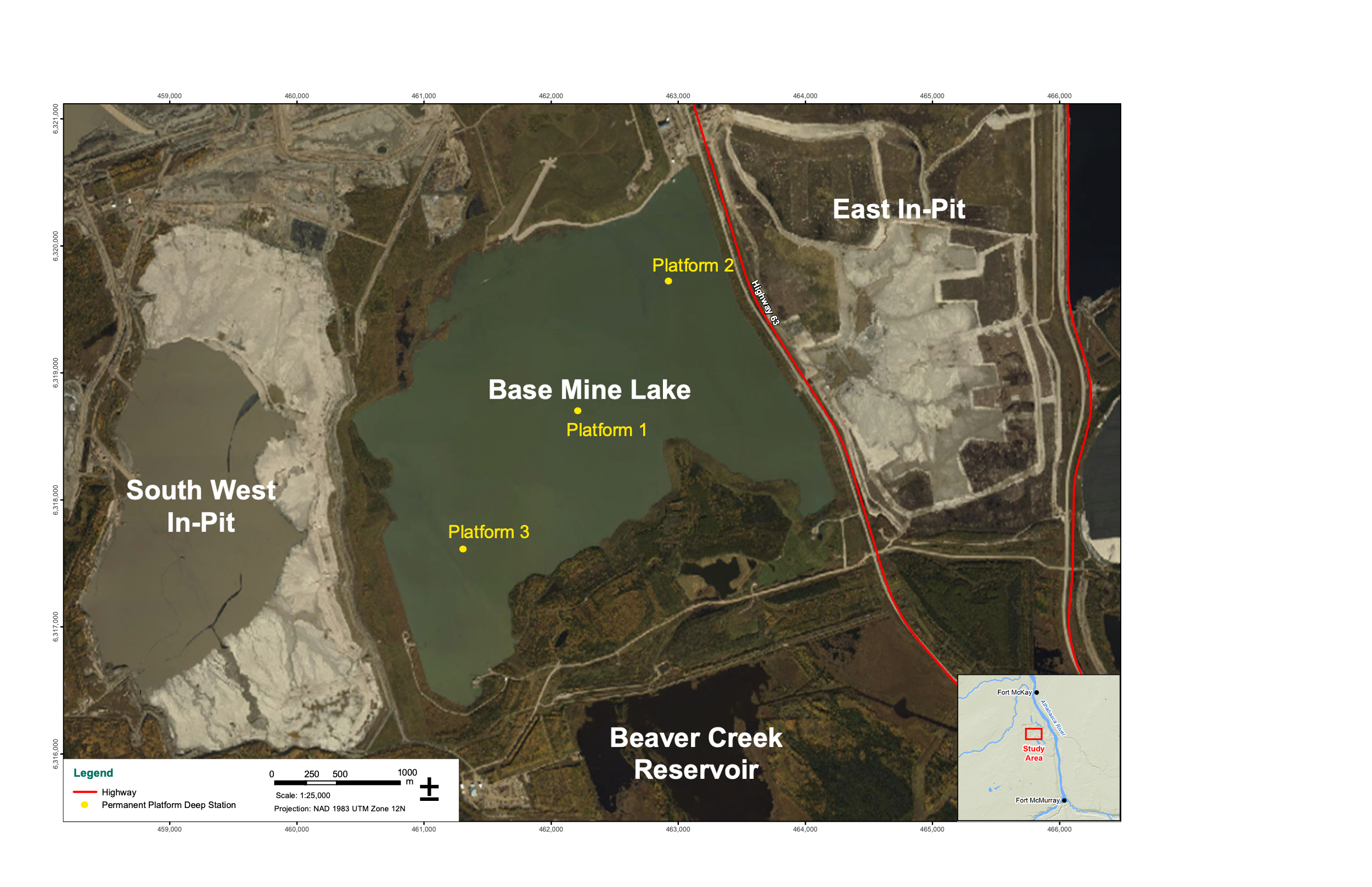


**Fig. S1:** Aerial view of Base Mine Lake, with sampling platforms indicated.


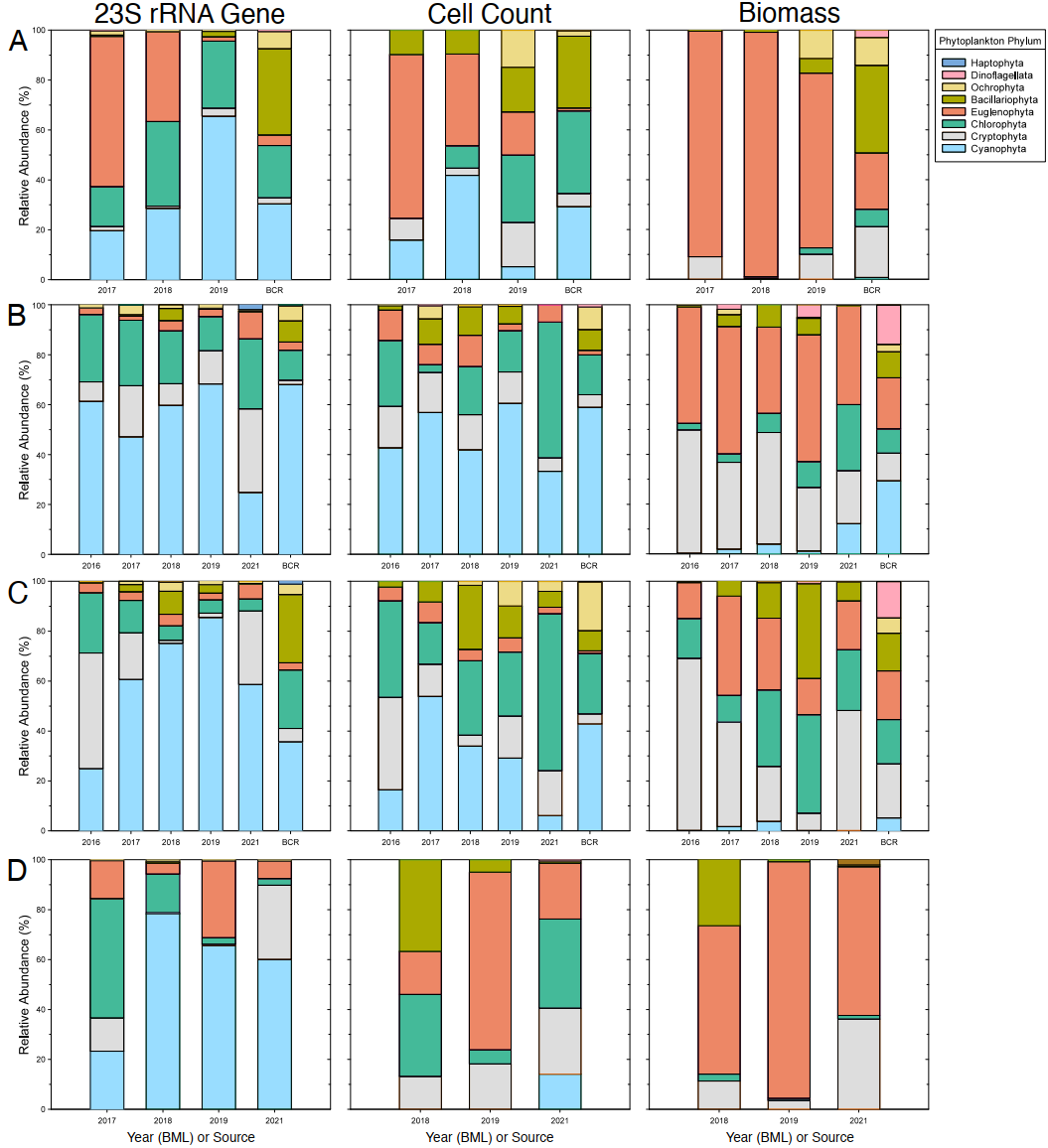


**Fig. S2**: Relative abundances of key phytoplankton phyla in surface waters of BML for each sampling year and for BCR (all years) based on 23S rRNA gene sequencing, cell count, and biomass data for spring (**A**), summer (**B**), autumn (**C**), and winter (**D**). No spring samples were taken in 2016 and 2021 in BML, and no winter samples were taken in 2016 or 2017 in BML or any year for BCR.


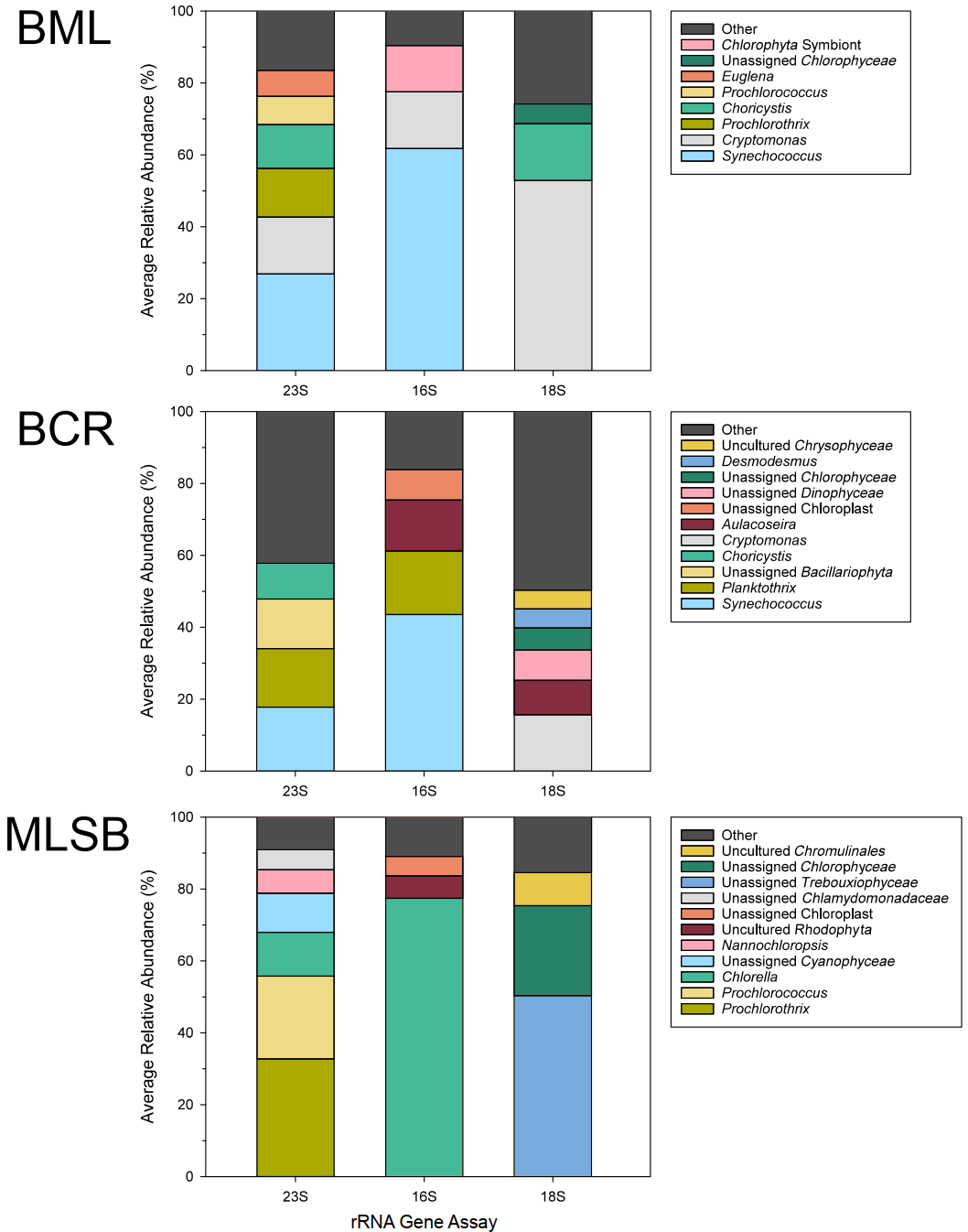


**Fig. S3**: Relative abundances of the most abundant (≥ 5%) phytoplankton genera in surface waters of BML, BCR, and MLSB for the 23S, 16S, and 18S rRNA genes.


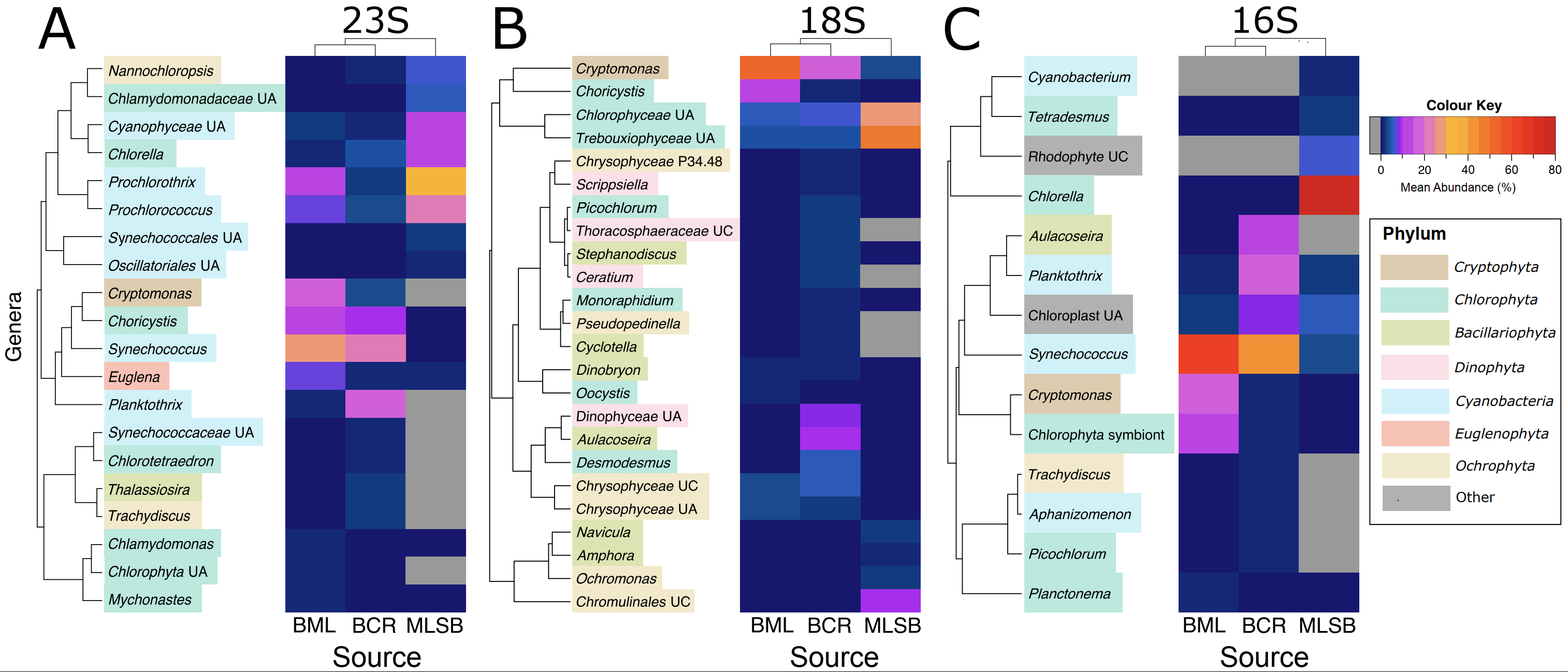


**Fig. S4**: Heatmaps depicting relative abundances of all genera comprising ≥ 1% of the total detected in the 23S (A), 16S (B), and 18S (C) rRNA gene sequencing analyses. Genera are colour-coded by phylum and clustered based on similarities across sites with the caveat that heatmaps are not based on shared sample dates.


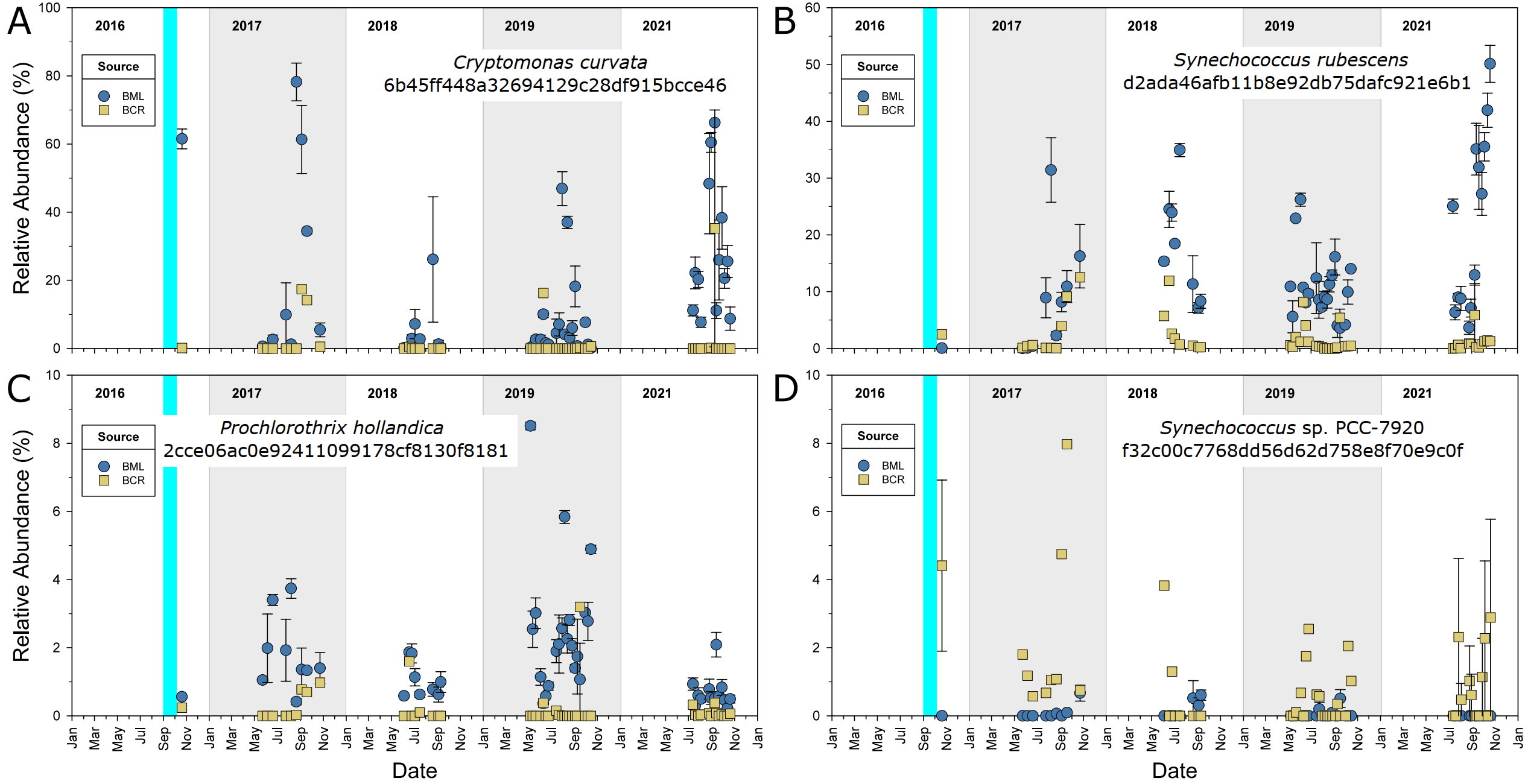


**Fig. S5**: Average relative abundance in BML and BCR surface waters over time (2016-2019, 2021) for four ASVs based on 23S rRNA gene data. Data are shared dates between BML and BCR and are means of three platforms ± 1 SEM, indicated by black bars. The teal bar indicates alum addition.

**
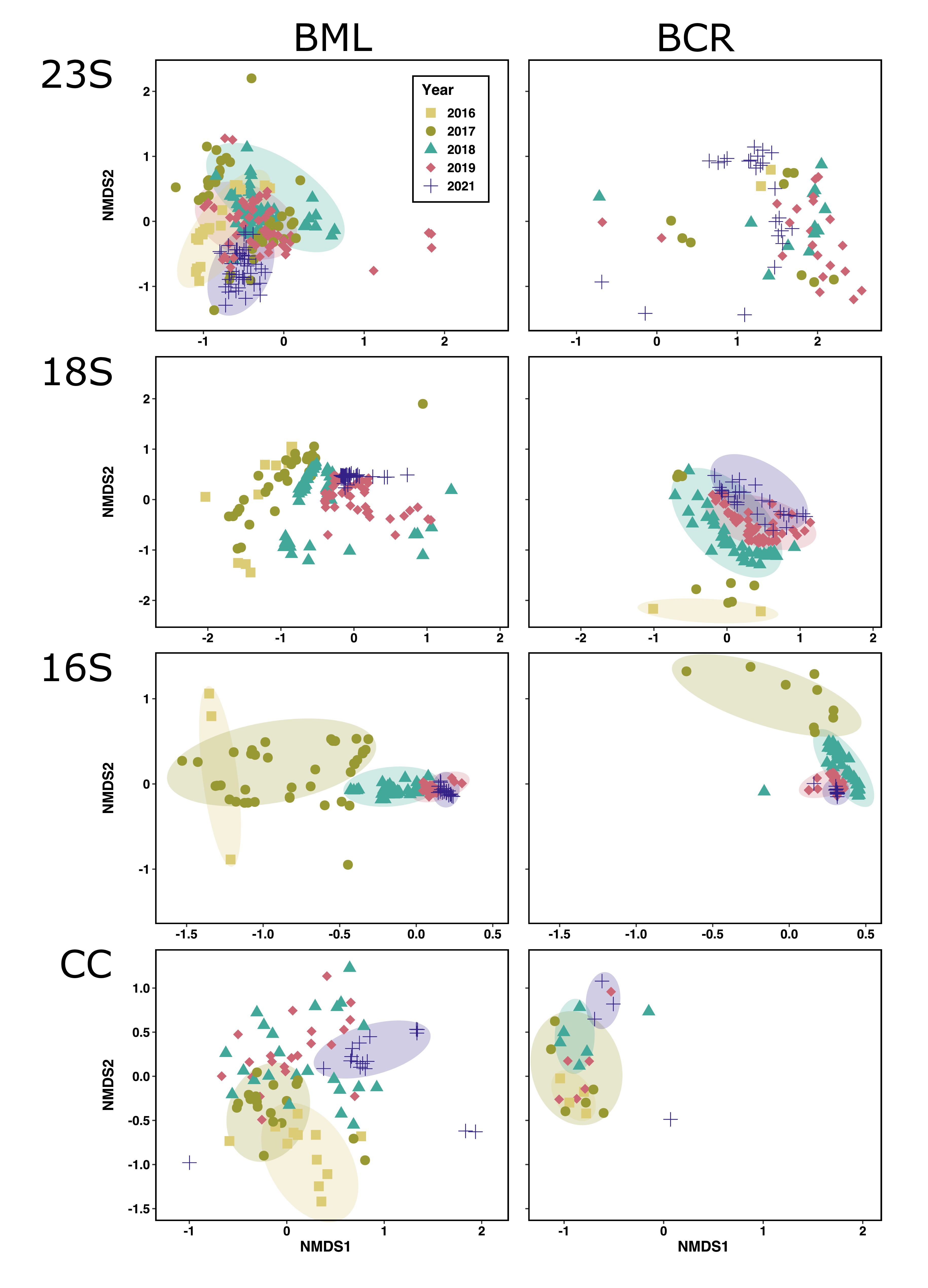
**

**Fig. S6**: Non-metric multi-dimensional scaling ordination (NMDS) plots based on Bray-Curtis distances of phytoplankton communities for the 23S, 16S, and 18S rRNA genes and for cell count data in BML and BCR surface waters by year. Data were normalized using scaling with ranked subsampling (SRS) [32], with counts normalized to 4000, 1000, 100, and 10000 for each dataset, respectively. For all plots, data are ASV-level and k = 2 axes. Stress scores are, respectively, 0.198, 0.207, 0.142, and 0.239. Clusters with ANOSIM support for that year are indicated with coloured circles (See Additional file 2: Table S16).


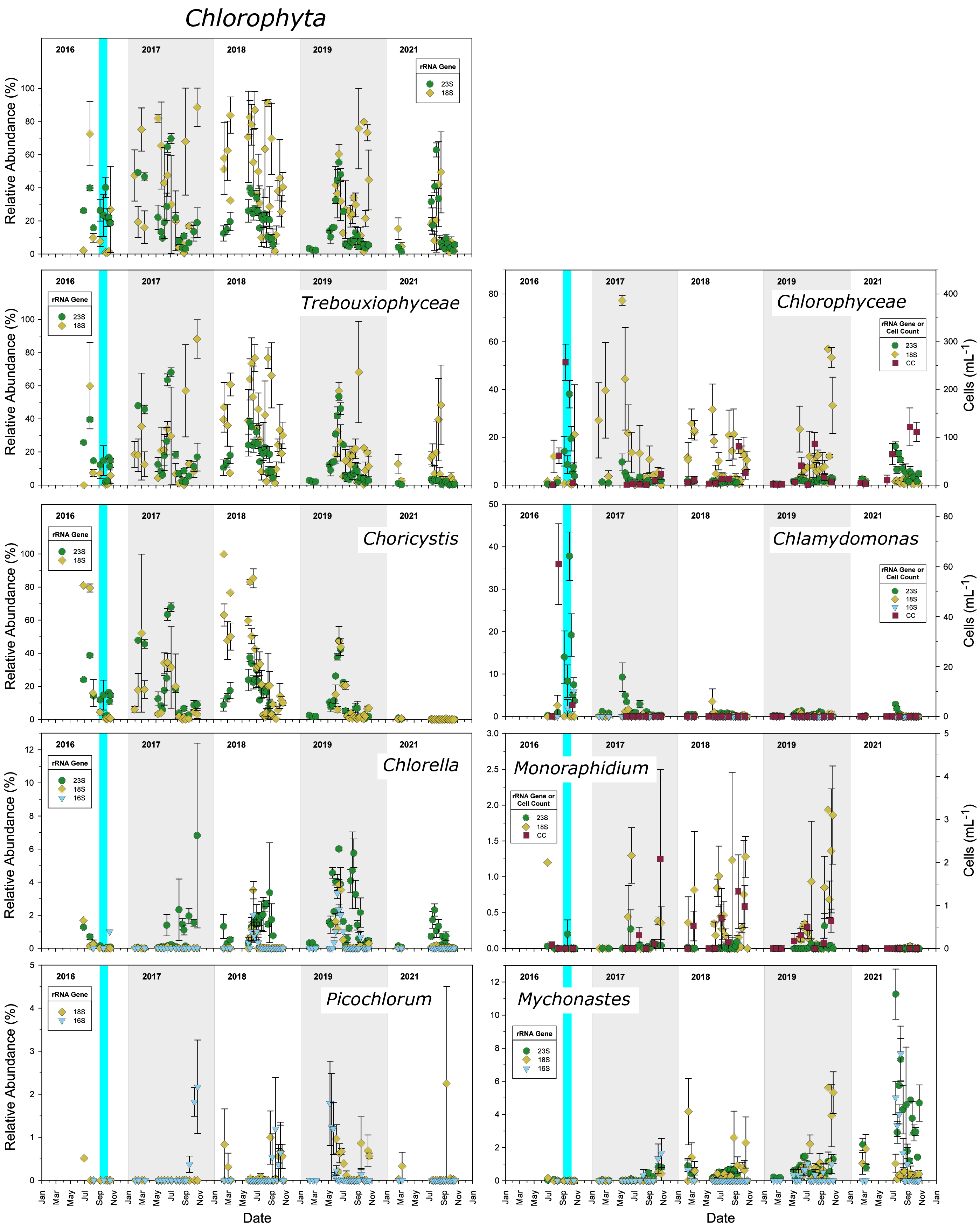


**Fig. S7:** Average relative abundance in BML surface waters over time (2016-2019, 2021) for key chlorophytes based on 23S, 16S, and 18S rRNA gene data with cell count data where applicable. The top panel contains the phylum *Chlorophyta* and below are the two main *Chlorophyta* families in BML (*Trebouxiophyceae* and *Chlorophyceae*). Below the family panels are genera belonging to those respective families. Data points are means of three platforms ± 1 SEM, indicated by black bars. The teal bar indicates alum addition. Cell count data were scaled to fit the patterns of the rRNA gene data.


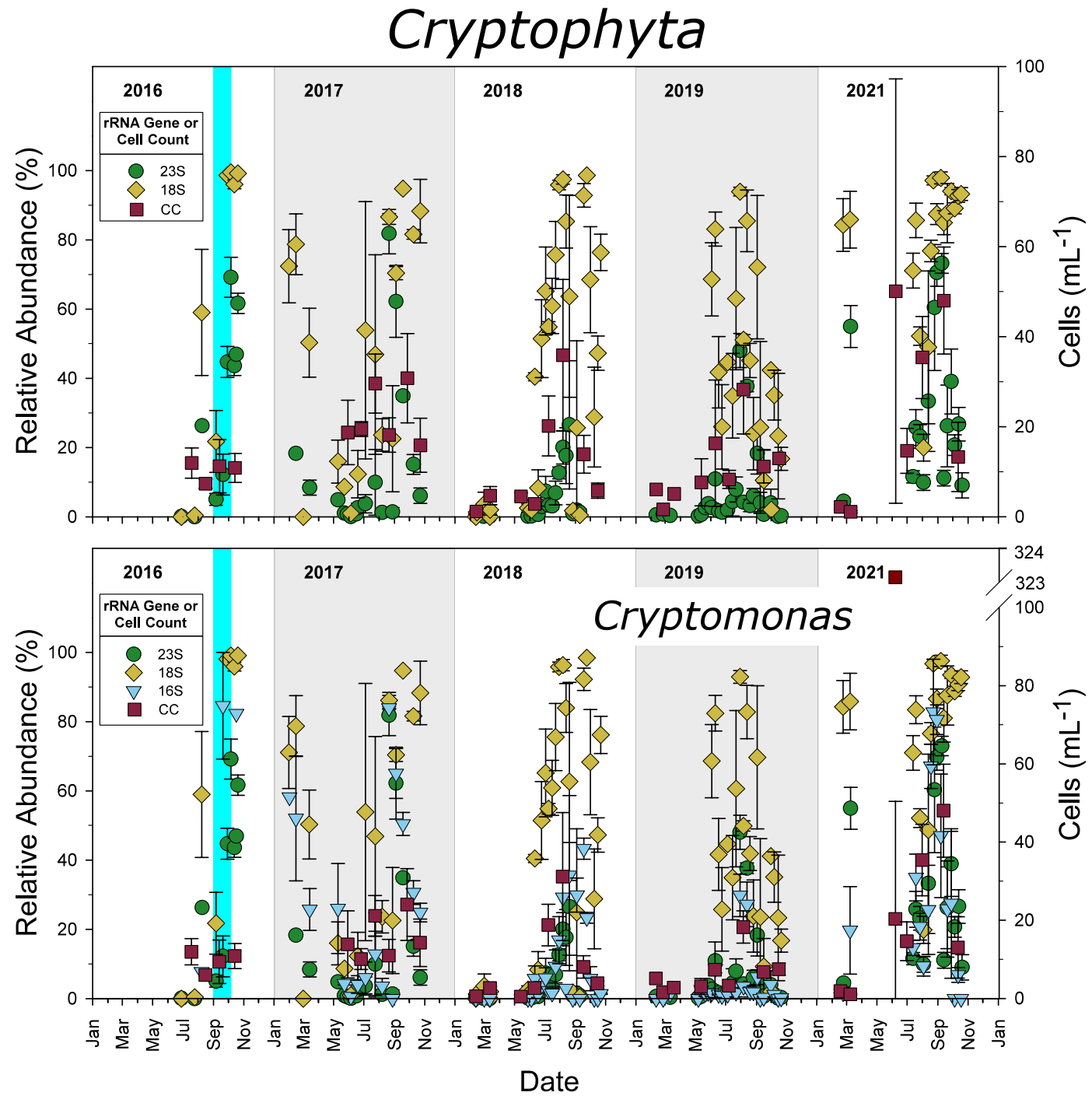


**Fig. S8:** Average relative abundance in BML surface waters over time (2016-2019, 2021) for the key cryptophyte *Cryptomonas* based on 23S, 16S and 18S rRNA gene data and cell count data. The top panel contains the phylum *Cryptophyta* and below is the main *Cryptophyta* genus in BML, *Cryptomonas*. Data points are means of three platforms ± 1 SEM, indicated by black bars. The teal bar indicates alum addition. Cell count data were scaled to fit the patterns of the rRNA gene data. No data for the 16S rRNA gene are plotted on the *Cryptophyta* graph because *Cryptomonas* was the only cryptophyte detected by 16S rRNA gene sequencing.


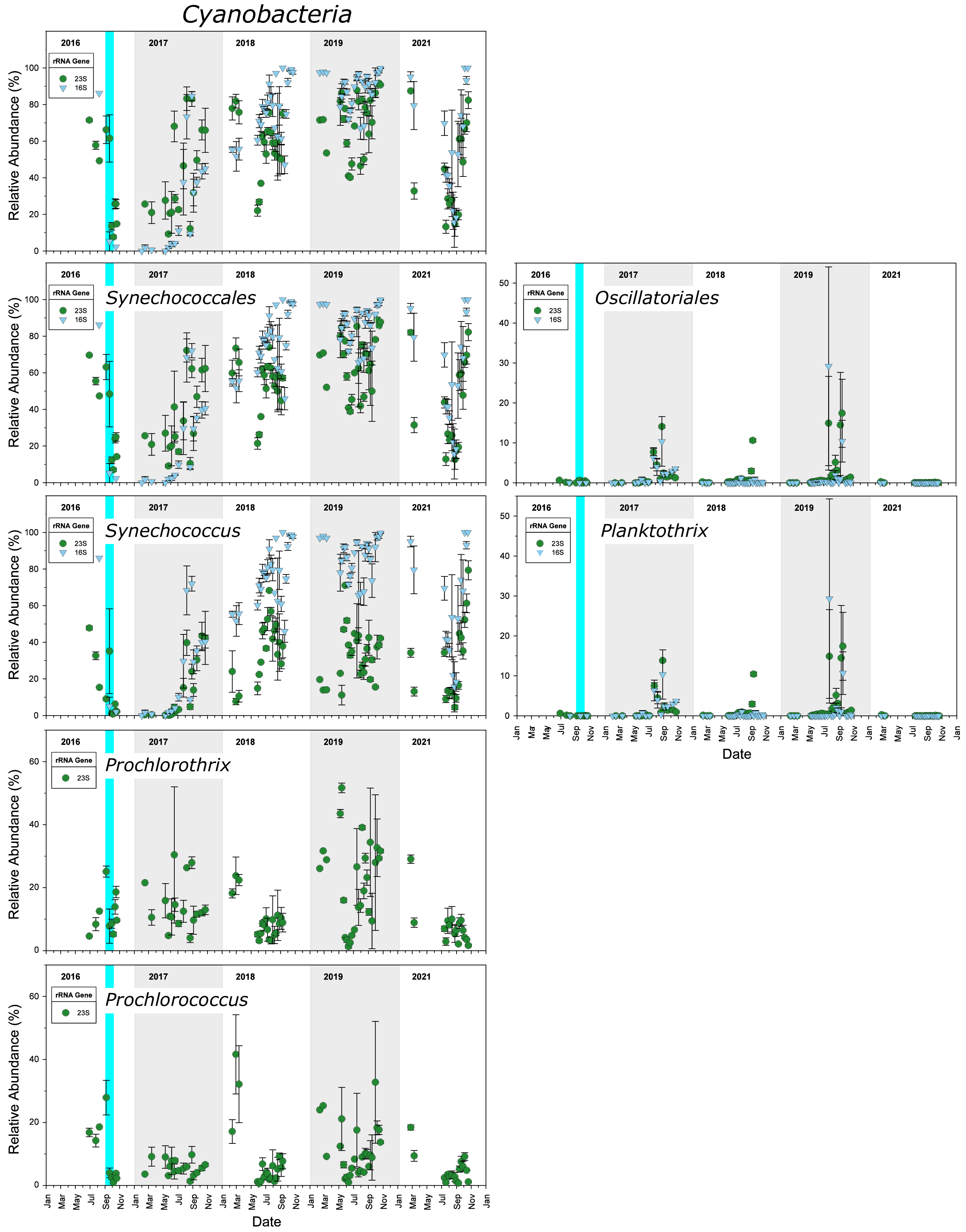


**Fig. S9A:** Average relative abundance in BML surface waters over time (2016-2019, 2021) for key cyanobacteria based on 23S and 16S rRNA gene data. The top panel contains the phylum *Cyanobacteria* and below are two main *Cyanobacteria* orders in BML (*Synechococcales* and *Oscillatoriales*). Below the order panels are *Cyanobacteria* genera belonging to those respective orders. Data points are means of three platforms ± 1 SEM, indicated by black bars. The teal bar indicates alum addition.

**
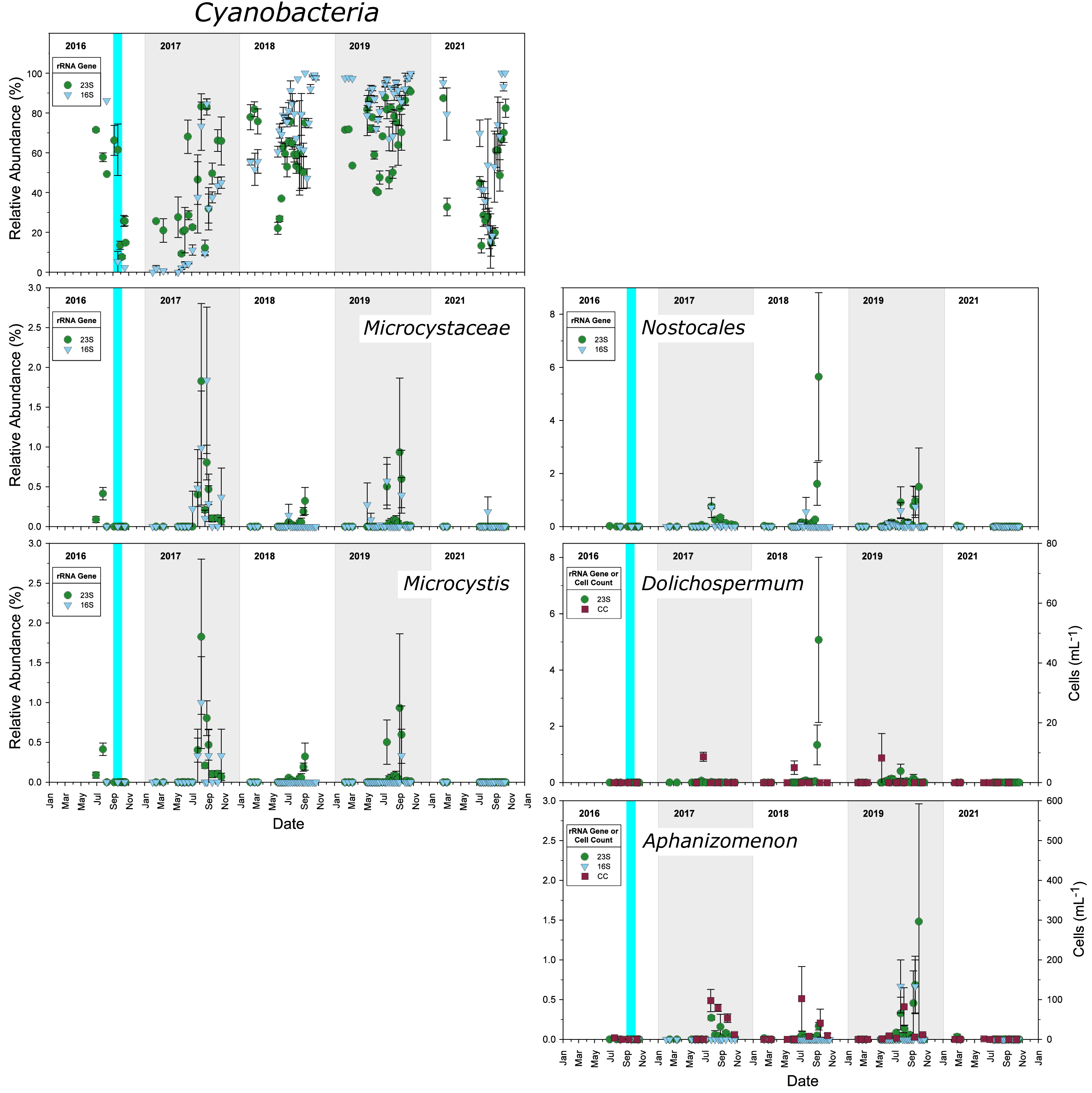
**

**Fig. S9B:** Average relative abundance in BML surface waters over time (2016-2019, 2021) for key cyanobacteria known to produce cyanotoxins based on 23S and 16S rRNA gene data and cell count data where applicable. The top panel contains the phylum *Cyanobacteria* and below are two main *Cyanobacteria* groups in BML (the family *Microcystaceae* and the order *Nostocales*). Below the group panels are *Cyanobacteria* genera belonging to those respective groups. Data points are means of three platforms ± 1 SEM, indicated by black bars. The teal bar indicates alum addition. Cell count data was scaled to fit the patterns of the rRNA gene data.


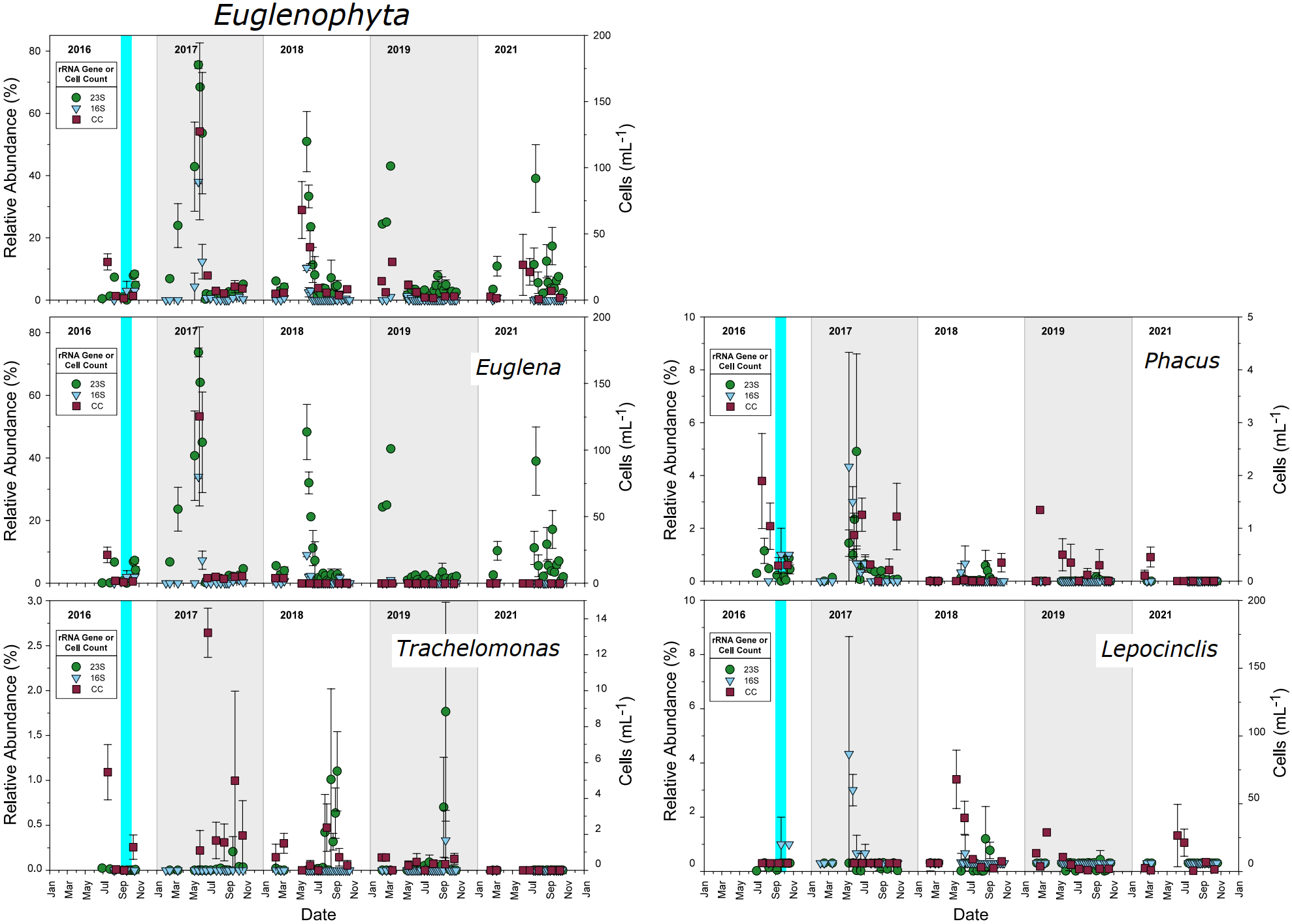


**Fig. S10:** Average relative abundance in BML surface waters over time (2016-2019, 2021) for key euglenophytes based on 23S, 16S, and 18S rRNA gene data and cell count data. The top panel contains the phylum *Euglenophyta* and below are the main *Euglenophyta* genera in BML. Data points are means of three platforms ± 1 SEM, indicated by black bars. The teal bar indicates alum addition. Cell count data were scaled to fit the patterns of the rRNA gene data.


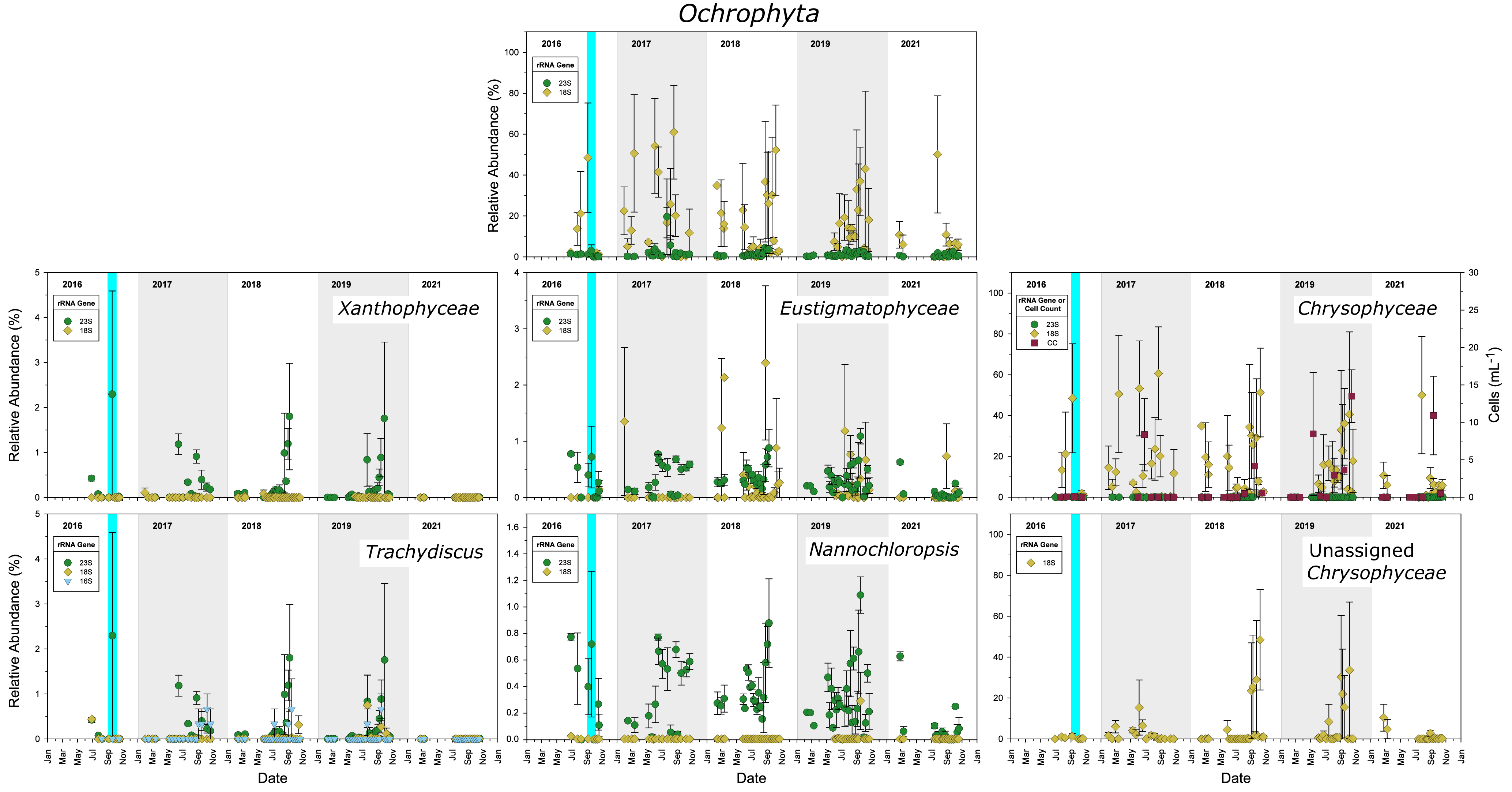


**Fig. S11:** Average relative abundance in BML surface waters over time (2016-2019, 2021) for key ochrophytes based on 23S and 18S rRNA gene data and cell count data where applicable. The top panel contains the phylum *Ochrophyta* and below are three main *Ochrophyta* families in BML (*Xanthophyceae, Eustigmatophyceae, and Chrysophyceae*). Below the family panels are genera belonging to those respective families. Data points are means of three platforms ± 1 SEM, indicated by black bars. The teal bar indicates alum addition. Cell count data were scaled to fit the patterns of the rRNA gene data.


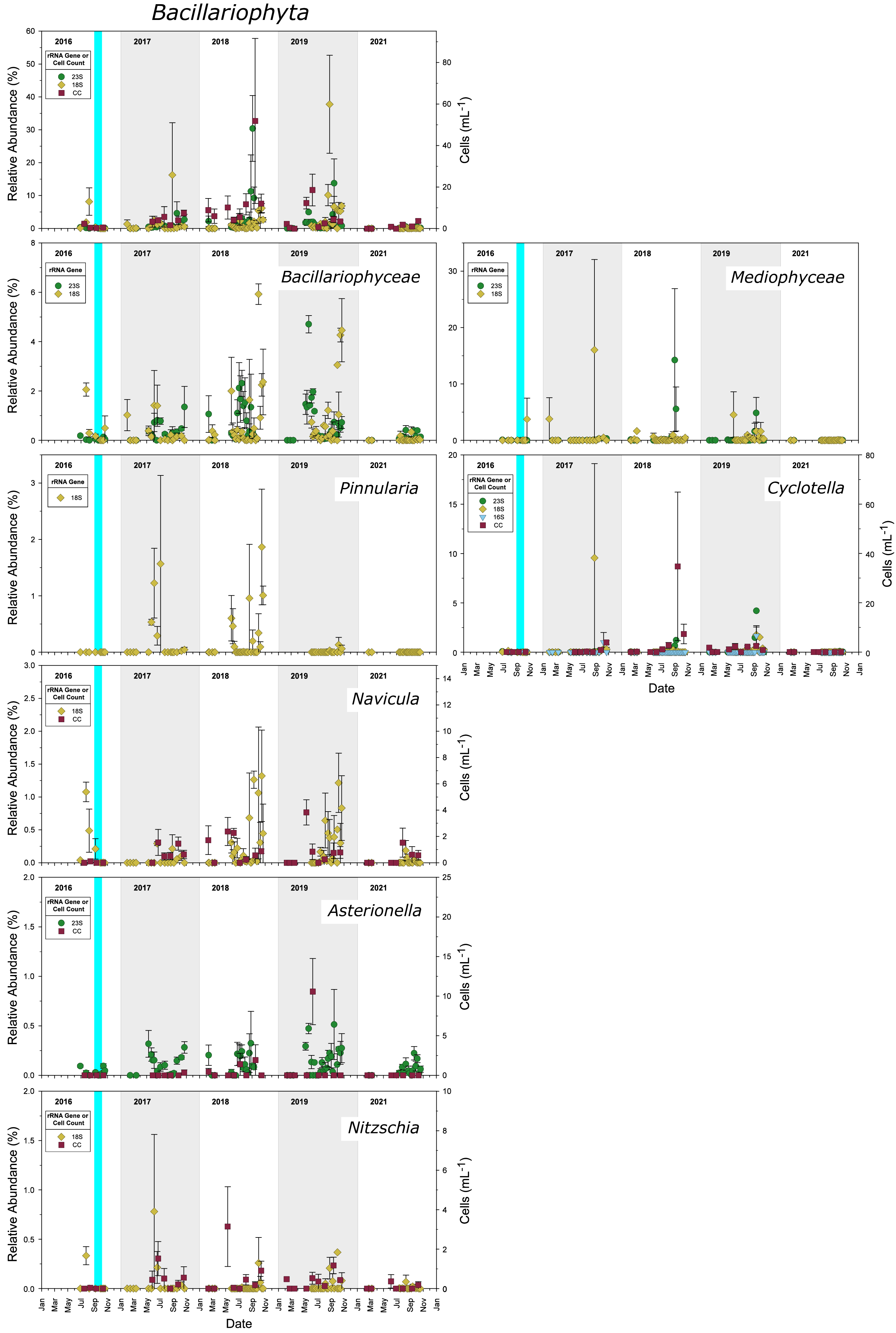


**Fig. S12A:** Average relative abundance in BML surface waters over time (2016-2019, 2021) for key bacillariophytes based on 23S, 16S, and 18S rRNA gene data and cell count data where applicable. The top panel contains the phylum *Bacillariophyta* and directly below are two main *Bacillariophyta* classes in BML (*Bacillariophyceae* and *Mediophyceae*). Below the class panels are genera belonging to those classes. Data points are means of three platforms ± 1 SEM, indicated by black bars. The teal bar indicates alum addition. Cell count data were scaled to fit the patterns of the rRNA gene data.


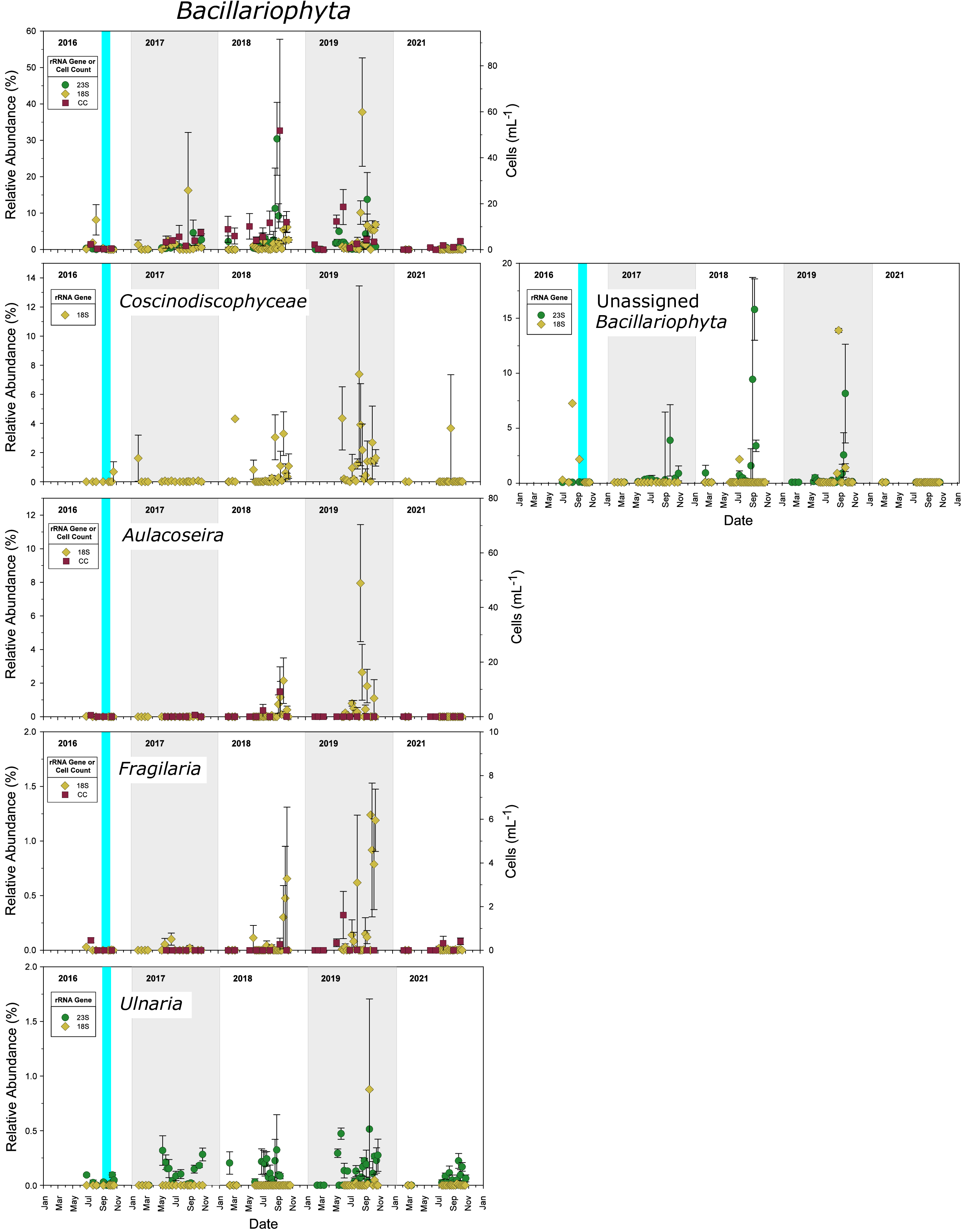


**Fig. S12B:** Average relative abundance in BML surface waters over time (2016-2019, 2021) for key bacillariophytes based on 23S, 16S, and 18S rRNA gene data and cell count data where applicable. The top panel contains the phylum *Bacillariophyta* and below are two main *Bacillariophyta* groups in BML (the class *Coscinodiscophyceae* and unassigned *Bacillariophyta*). Below the *Coscinodiscophyceae* panel are genera belonging to that class. Data points are means of three platforms ± 1 SEM, indicated by black bars. The teal bar indicates alum addition. Cell count data were scaled to fit the patterns of the rRNA gene data. *Note: *Ulnaria* was grouped under *Bacillariophyceae* for the 23S rRNA gene and *Coscinodiscophyceae* (*Fragillariophyceae*) for the 18S rRNA gene.

**
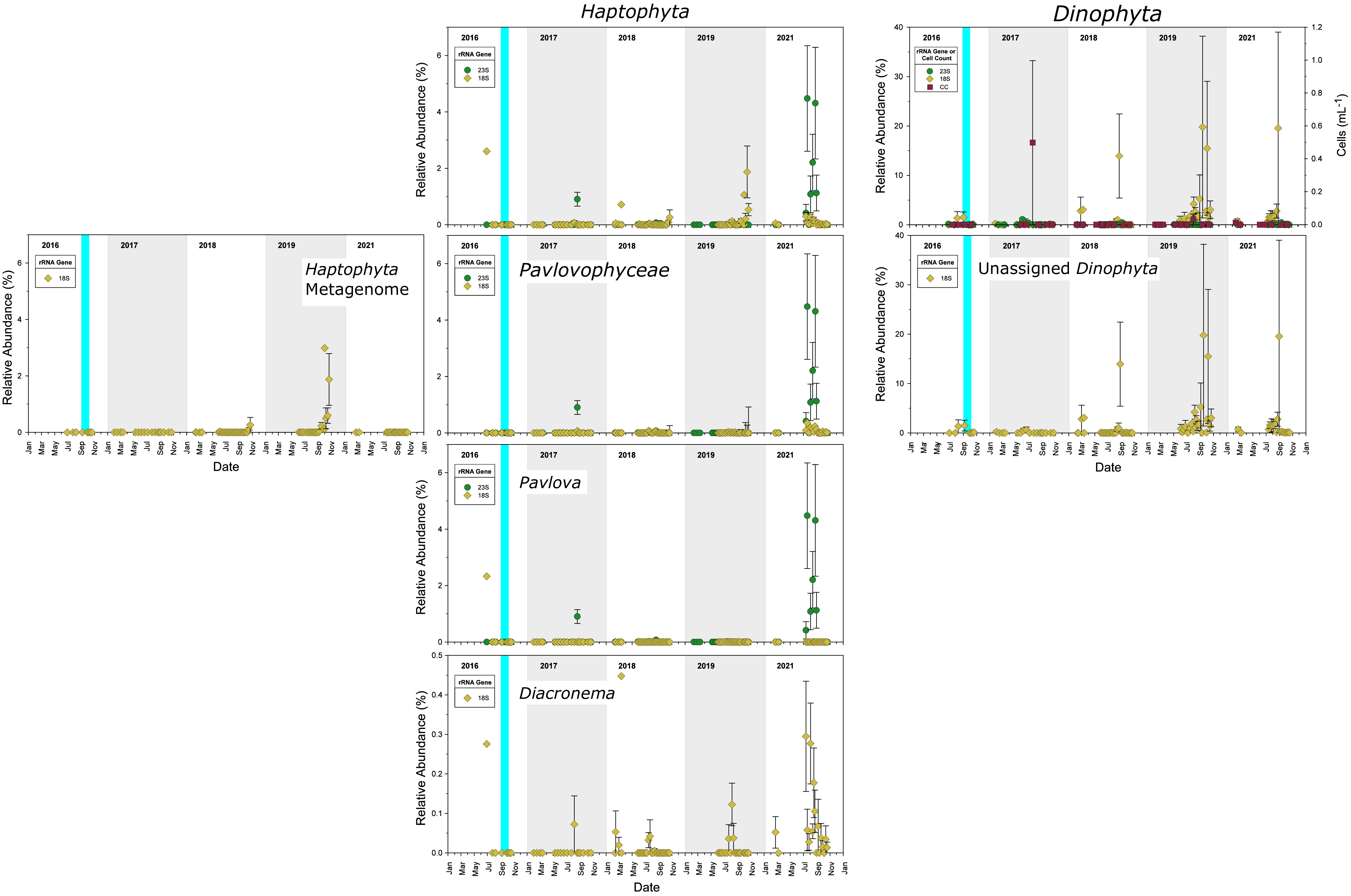
**

**Fig. S13:** Average relative abundance in BML surface waters over time (2016-2019, 2021) for key Haptophytes and Dinophytes based on 23S and 18S rRNA gene data and cell count data where applicable. The top panels contain the phyla *Haptophyta* (left) and *Dinophyta* (right). Below the *Haptophyta* panel are two main *Haptophyta* groups in BML (*Haptophyta* Metagenome on the left and the family *Pavlovophyceae* on the right). Below *Pavlovophyceae* are main genera in BML belonging to that family. Below *Dinophyta* is the main *Dinophyta* OTU found in BML, Unassigned *Dinophyta*. Data points are means of three platforms ± 1 SEM, indicated by black bars. The teal bar indicates alum addition. Cell count data were scaled to fit the patterns of the rRNA gene data.


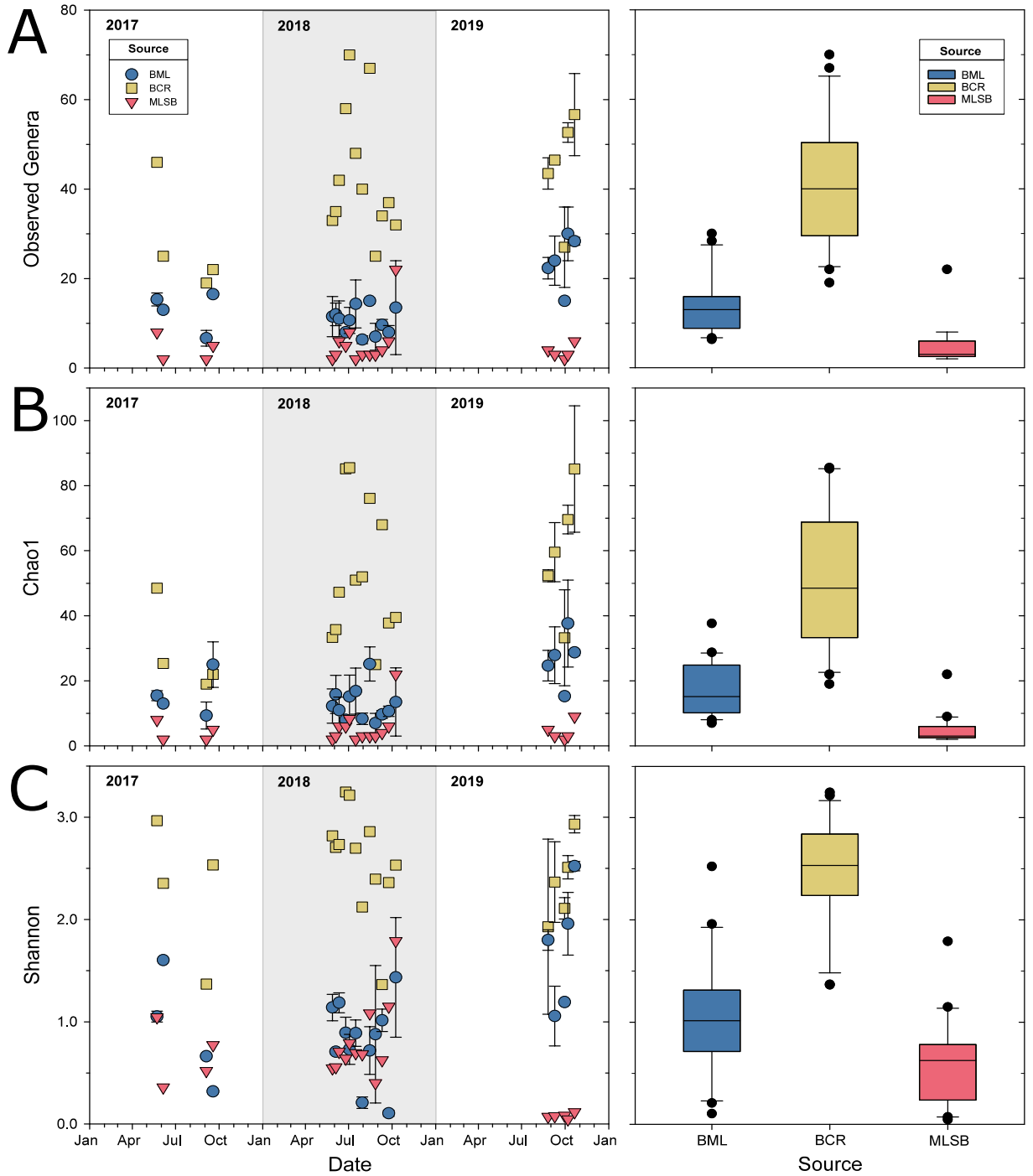


**Fig. S14**: Alpha-diversity indices in BML, BCR, and MLSB surface waters on shared sample dates, based on the 18S rRNA gene sequencing at the genus level, normalized to 1000 counts using scaling with ranked subsampling (SRS) and filtered to include only phytoplankton. The 18S rRNA gene was chosen because it had the most shared sample points. Data points are means of three platforms ± 1 SEM. Stem-and-leaf plots (right) for each index indicate means, 95% confidence intervals, and ranges averaged by site over all sampling dates; points indicate outliers.


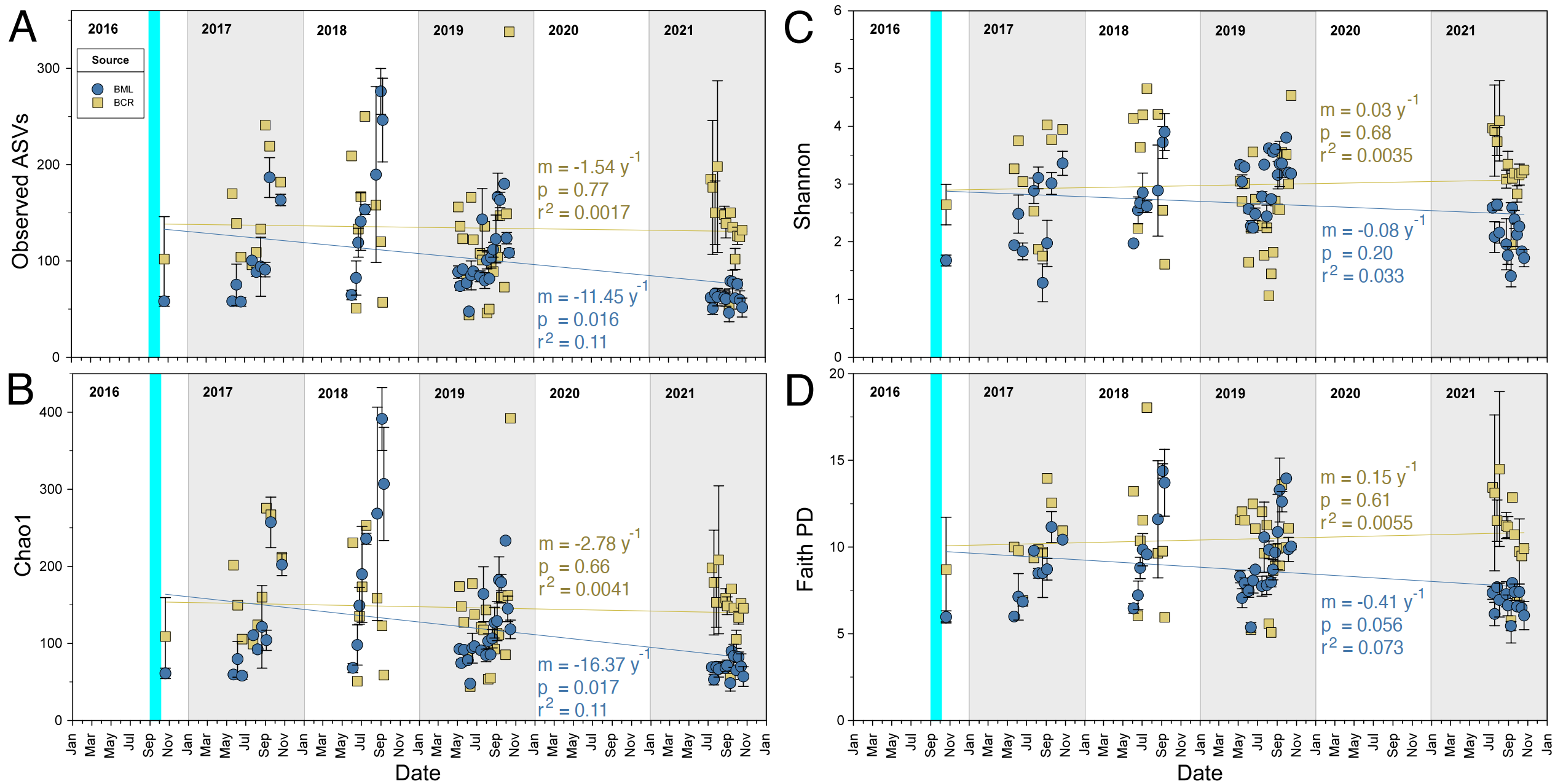


**Fig. S15**: Alpha diversities over time with regression lines comparing BML to BCR surface waters for the 23S rRNA gene, accounting for both eukaryotic algae and cyanobacteria. Data are identified to the ASV level and normalized to 4000 counts using scaling with ranked subsampling (SRS) [32]. Given are the slope (m) per year, p-values based on a t-test of slope=0, and r^2^ value for the regression lines. Data points are means of three platforms ± 1 SEM. The teal bar indicates alum addition.


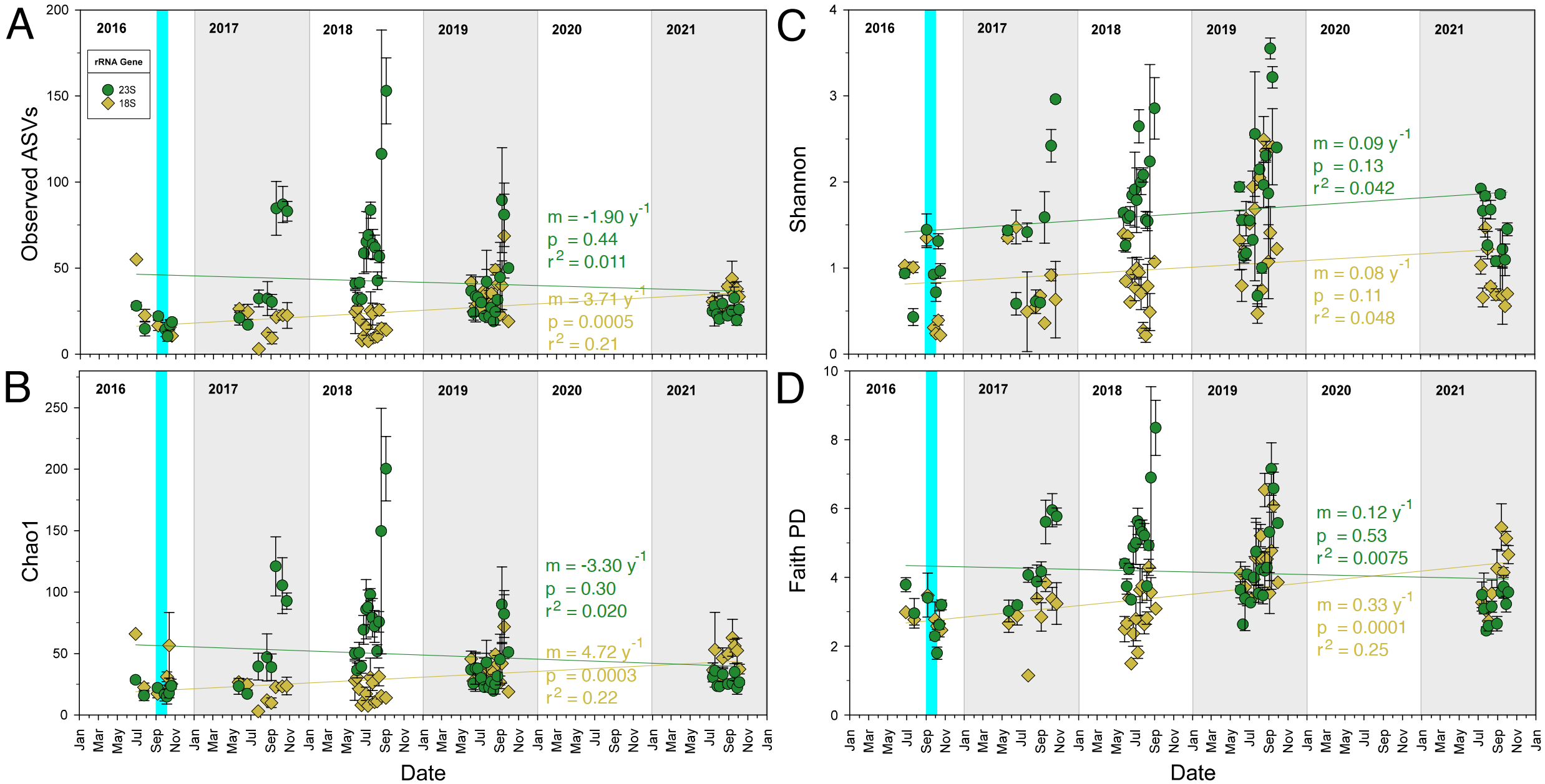


**Fig. S16:** Alpha diversities over time in BML surface waters, for datasets filtered to include only eukaryotic algae. 18S rRNA gene and 23S rRNA gene data were filtered for eukaryotes at the genus level and both normalized to 2000 counts using scaling with ranked subsampling (SRS). Given are the slope (m) per year, p-values based on a t-test of slope=0, and r^2^ value for the regression lines. Data points are means of three platforms ± 1 SEM, indicated by black bars. The teal bar indicates alum addition.
